# The Role of IL-33/ST2 Pathway in Tumorigenesis

**DOI:** 10.3390/ijms19092676

**Published:** 2018-09-09

**Authors:** Kristen M. Larsen, Maydelis Karla Minaya, Vivek Vaish, Maria Marjorette O. Peña

**Affiliations:** 1 Department of Biological Sciences, University of South Carolina, Columbia, SC 29208, USA; kmlarsen@email.sc.edu (K.M.L.); maydelis_minaya@brown.edu (M.K.M.); vivekvaish@live.com (V.V.); 2 Department of Biological Sciences and Center for Colon Cancer Research, University of South Carolina, Columbia, SC 29208, USA

**Keywords:** Interleukin 33, IL-33/ST2 signaling, tumor microenvironment, inflammation, cancer

## Abstract

Cancer is initiated by mutations in critical regulatory genes; however, its progression to malignancy is aided by non-neoplastic cells and molecules that create a permissive environment known as the tumor stroma or microenvironment (TME). Interleukin 33 (IL-33) is a dual function cytokine that also acts as a nuclear factor. IL-33 typically resides in the nucleus of the cells where it is expressed. However, upon tissue damage, necrosis, or injury, it is quickly released into extracellular space where it binds to its cognate receptor suppression of tumorigenicity 2 (ST2)L found on the membrane of target cells to potently activate a T Helper 2 (Th2) immune response, thus, it is classified as an alarmin. While its role in immunity and immune-related disorders has been extensively studied, its role in tumorigenesis is only beginning to be elucidated and has revealed opposing roles in tumor development. The IL-33/ST2 axis is emerging as a potent modulator of the TME. By recruiting a cohort of immune cells, it can remodel the TME to promote malignancy or impose tumor regression. Here, we review its multiple functions in various cancers to better understand its potential as a therapeutic target to block tumor progression or as adjuvant therapy to enhance the efficacy of anticancer immunotherapies.

## 1. Introduction

Cancer is a major public health concern worldwide and is the second leading cause of deaths in the United States after heart disease [1]. In 2018, an estimated 1.7 million new cases will be diagnosed and approximately 609,640 patient deaths are predicted to occur [2]. While cancer initiation and progression to malignancy is fundamentally driven by mutations in key regulatory genes, genomic instability, epigenetic changes, and chromosomal alterations [3], the impact of non-neoplastic cells in the tumor microenvironment (TME) on tumor progression has been firmly established [3,4,5]. Residing within a tumor are a heterogeneous population of neoplastic cells with varying capabilities for invasiveness, malignancy, and sensitivity to anti-tumor therapies, and an equally heterogeneous collection of non-malignant cellular and non-cellular components that collectively make up the tumor stroma or microenvironment [3,5]. These non-neoplastic cells are recruited by molecular signals from tumor cells into the TME, where they secrete a diverse combination of cytokines, growth factors, hormones and reactive oxygen species that activate signaling pathways that promote tumor growth and development [5]. Indeed, tumor angiogenesis, progression to malignancy, and metastasis are dependent on tumor interactions with these stromal cells.

Interleukin 33 (IL-33) is a member of the IL-1 family of cytokines that is expressed in multiple organs and cell types in humans and mice [6] and is the ligand for the suppression of tumorigenicity 2 (ST2)L receptor. IL-33/ST2 signaling has been shown to potently invoke a T Helper 2 (Th2) immune response in inflammatory diseases and wound healing responses in several tissues, and has been implicated in many inflammatory and allergic diseases [7,8] including rheumatoid arthritis [9], atopic dermatitis [10], asthma [11], inflammatory bowel disease [12], and cardiovascular disease [13,14], among others. While the role of IL-33/ST2 has been widely studied and established in inflammatory diseases [7,15], its role in cancer is yet to be fully elucidated. Here, we review the sometimes opposing roles of the IL-33/ST2 axis as studied thus far in different types of cancers. Although IL-33 has been reported to play pro-tumorigenic or anti-tumorigenic roles in cancer development, both functions implicate IL-33 as a potent modulator of the TME by playing a major role in recruiting immune cells that impact tumor phenotype and malignancy. For each type of cancer, we discuss the pro-tumorigenic role of IL-33/ST2 signaling, and, unless discussed in historic terms, this is followed by its anti-tumorigenic role in cases where opposing roles have been reported. We examine the impact of these observations on targeting IL-33/ST2 in therapeutic applications for the treatment or management of cancer progression. We begin our discussion with breast cancer where the role of IL-33 in tumorigenesis was first identified, followed by colorectal cancer where many studies have been conducted, and then discuss each cancer based on their organ of origin and grouping according to the human organ systems. In the concluding section, we summarize the observed roles of IL-33/ST2 signaling in the different types of cancers, the experimental models used to establish its role and the corresponding references as a quick guide on the progress thus far in our understanding of its impact on tumor progression and therapeutic applications.

## 2. The IL-33/ST2 Signaling Pathway

### 2.1. Interleukin 33

IL-33 was identified as a member of the IL-1 family of cytokines in 2005 and the ligand for what was thought to be an orphan receptor, ST2L [6]. It is constitutively expressed in many tissues and by a wide variety of cells. However, it is also induced in response to various stimuli in epithelial cells, myofibroblasts, adipocytes, endothelial cells, smooth muscle cells, and macrophages predominantly as a pro-inflammatory cytokine [16,17]. IL-33 is a 30-kDa protein that functions dually as a transcription factor and a cytokine. Its N-terminus contains a nuclear localization signal, a DNA-binding homeodomain-like helix-turn-helix motif, and a chromatin binding domain [6,18], while the C-terminus contains an IL-1 like cytokine domain [6]. Full length IL-33 is targeted to the nucleus upon synthesis, where it binds to chromatin and is thought to regulate gene expression by a number of mechanisms. It can bind to histones H2A and H2B [18,19] and can activate histone deacetylase-3 (HDAC) activity [20] thereby affecting gene expression by remodeling chromatin structure and by epigenetic mechanisms. It has been shown to interact with the N-terminal domain of the p65 subunit of nuclear factor κB (NF-κB) to repress the expression of NF-κB-regulated genes that are necessary for pro-inflammatory signaling [21]. In response to cellular damage, tissue injury or viral infection, IL-33 is quickly released from the nucleus of necrotic cells and secreted into extracellular space where it can bind to the membrane-bound ST2L receptor through its cytokine domain [22,23]. Binding to its receptor triggers an inflammatory cascade, thus, IL-33 acts as an “alarmin” and is considered a damage-associated molecular pattern (DAMP) [24]. The nuclear and cytokine functions of IL-33 are tightly regulated through its localization [7]. Transgenic mice expressing *IL-33* where nuclear targeting had been abolished succumbed to lethal inflammation resulting from the uncontrolled influx of eosinophils and other inflammatory cells into multiple organs [25]. Thus, full length nuclear IL-33 acts as a transcription factor that modulates cytokine gene expression and its nuclear compartmentalization is a deterrent to unleashing damaging inflammation instigated by its alarmin and cytokine functions [7].

**Figure 1:**
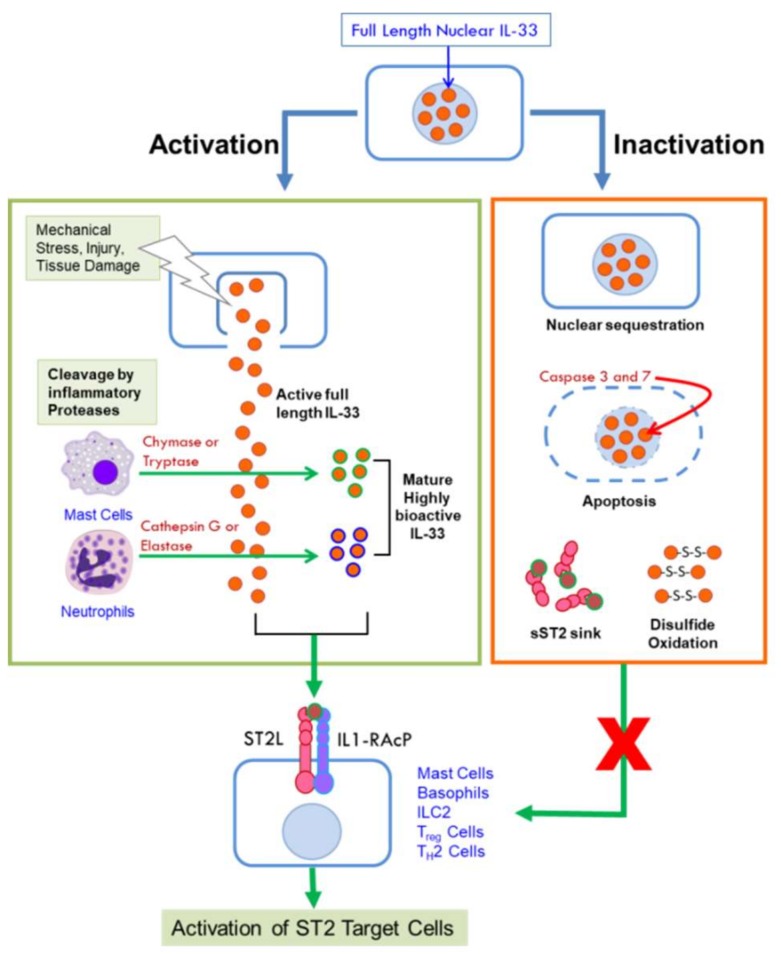
Mechanisms of modulating IL-33 localization and cytokine function. Upon synthesis, full length (FL)-IL-33 is targeted to the nucleus by a nuclear localization signal where it binds to chromatin to repress expression of inflammatory genes. **Activation**. In response to mechanical stress, tissue injury or damage, or necrosis, IL-33 is released into extracellular space by a poorly understood mechanism. Under inflammatory conditions, FL-IL-33 is active, but its activity is amplified upon cleaved by proteases secreted by neutrophils (cathepsin G or elastase) or mast cells (chymase or tryptase) to generate truncated forms that are 10–30-fold more bioactive. FL-IL-33 and its derivatives then bind to the ST2L receptor expressed on the cell surface of target cell. **Inactivation**. Under homeostatic conditions, sequestration of IL-33 in the nucleus prevents the unleashing of its cytokine function. During apoptosis, IL-33 is inactivated by the apoptotic proteases (caspase 3 and 7) to prevent its activation of the immune response upon apoptotic release. Extracellular IL-33 can be scavenged by the soluble decoy receptor sST2 or oxidized at cysteine residues to form disulfide bonds to block its interaction with ST2L receptor. Green arrows indicate activation while red arrow indicates inactivation of IL-33 or IL-33/ST2 signaling.

Full-length (FL) IL-33 can function as a cytokine, unlike other IL-1 cytokine family members IL-1β and IL-18 that require cleavage by caspase-1 for biological activity [6]. Further studies showed that IL-33 is not a substrate for caspase-1 [23,26] but is degraded by the pro-apoptotic caspases 3 and 7 [27,28] resulting in its inactivation. Thus, rather than a pre-requisite for its activation, cleavage by these caspases is thought to act as a switch to extinguish the pro-inflammatory activity of IL-33 [28], ensuring immune tolerance during apoptosis by preventing its secretion. On the other hand, under inflammatory conditions, full length human and mouse IL-33 are cleaved by the serine proteases cathepsin G and elastase released by neutrophils to generate mature forms that are ten-fold more bioactive than FL-IL-33 [29]. IL-33 can also be cleaved by chymase and tryptase proteases secreted by activated mast cells, critical effector cells in allergic disorders, to potently activate group 2 innate lymphoid cells [30]. Cleavage by mast cell proteases generate three different mature isoforms of IL-33 that are 30-fold more bioactive than FL-IL-33 [30]. It is important to note that both mast cells and neutrophils are abundantly recruited into the TME. The mature forms of IL-33 lack the N-terminal domain and function as IL-1-like cytokines through their C-terminal domain. Thus, the activity of IL-33 can be amplified in the context of an inflammatory microenvironment through the action of proteases secreted by innate cells that are recruited in response to injury or inflammation [29,30,31]. Figure 1 summarizes the mechanisms by which the cytokine activity of IL-33 is unleashed or diminished.

### 2.2. The ST2 Receptor

The ST2 receptor had been extensively studied prior to the discovery of its ligand IL-33. Suppression of tumorigenicity 2 (ST2) was first identified in murine fibroblasts as an oncogene-induced gene [32,33]. It is encoded by *IL1RL1* that produces four isoforms through alternative splicing: ST2L (ligand), sST2, ST2V (variant), and ST2LV (ligand variant). ST2L is a membrane embedded receptor that is highly homologous to IL-1 type-1 receptors and harbors three Ig-like extracellular domains, a transmembrane spanning region, and an ILI-R1-like intracellular domain [34,35]. sST2 is a soluble form of ST2 that is secreted as a glycosylated protein. It lacks the transmembrane domain but contains an extracellular domain similar to ST2L and an additional nine amino acids at the C-terminal tail [34,36]. ST2V is similar to sST2 but contains a hydrophobic tail in place of the third Ig-like domain [37,38]. ST2LV is a soluble and *N*-glycosylated isoform that lacks the transmembrane domain found in ST2L [39]. Of the four isoforms, ST2L and its decoy receptor sST2 have been most studied, while not much is known about ST2V and ST2LV. ST2L forms a heterodimeric transmembrane receptor complex with the IL1-receptor accessory protein, IL1-RAcP that is necessary for signal transduction upon binding of IL-33 [36,40], while sST2 functions to sequester extracellular IL-33. Expression of the *IL1RL1* gene was regulated by GATA1/2, and estrogen-response elements (EREs) are found on the distal and proximal promoters that regulate ST2L and sST2 expression [36,41,42]. ST2L is expressed in fibroblasts, mast cells, eosinophils, Th2 lymphocytes, dendritic cells, basophils, invariant natural killer cells (iNKT) cells, and macrophages, while sST2 is predominantly expressed by fibroblasts and epithelial cells ([43] and references therein). While both isoforms can be transcribed from both promoters, the cell type appears to govern gene expression from either proximal or distal promoters [41].

### 2.3. Signaling by the IL-33/ST2 Pathway

**Figure 2:**
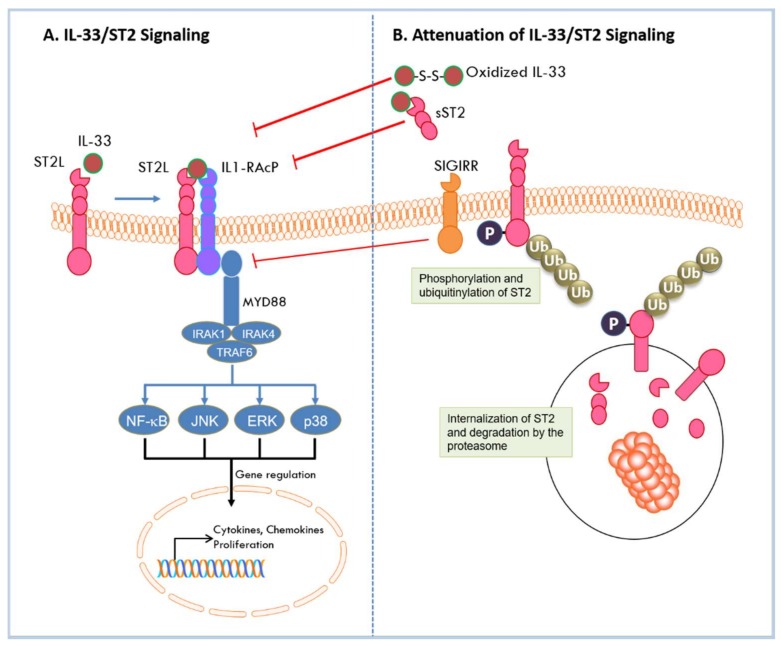
Activation and Attenuation of IL-33/ST2 Signaling. (**A**) IL-33/ST2 Signaling. IL-33 binds to ST2L causing a conformational change that leads to recruitment of IL1-RAcP. Heterodimerization of the transmembrane proteins results in interaction between their intracellular C-terminal domains that facilitates recruitment of adaptor molecules including MyD88, IRAK1, IRAK4, and TRAF6. Subsequent activation of transcription factors NF-κB, JNK, ERK, and p38 leads to expression of genes encoding cytokines, chemokines, and growth factors. (**B**) Attenuation of IL33-ST2 signaling. SIGIRR can disrupt the ST2L/IL1RAcP heterodimer. Phosphorylated ST2L is quickly internalized, polyubiquitinylated by the E3 ligase FBXL19 and targeted for degradation by the proteasome. Extracellular IL-33 can be sequestered by sST2 acting as a molecular decoy and can be quickly oxidized via its cysteine residues. Both processes prevent its interaction with the membrane bound ST2L receptor. Blue arrows indicate interaction between molecules, black arrows indicate gene regulation or transcriptional activation; red lines with bars indicate disruption of molecular interactions.

As a nuclear factor, IL-33 binds to chromatin to repress the expression of inflammatory responses. As a cytokine, IL-33 is secreted into extracellular space in response to cell damage or mechanical injury [44]. IL-33 can then bind to the ST2L receptor, via its C-terminal IL-1 like cytokine domain, inducing a conformational change that results in recruitment of IL-1RAcP to form a heterodimeric receptor complex on the cell membrane [23]. Hetero-dimerization brings together the intracellular domains of the two transmembrane proteins, and its assembly initiates the recruitment of adaptor molecules through which the IL-33 signal is transduced. These molecules include the myeloid differentiation primary response protein 88 (MyD88), IL-1 receptor-associated kinases (IRAK-1 and IRAK-4), and tumor necrosis factor (TNF) acceptor associated factor 6 (TRAF6) [6,45]. This leads to the degradation of inhibitory protein IκB and subsequent activation of NF-κB transcription factor. In addition, activation of mitogen activated protein (MAP) kinases p38, c-Jun N-terminal kinase (JNK), and extracellular signal-regulated kinase (ERK) results in the activation of downstream transcription factors such as activator protein (AP)-1 [7]. These transcription factors direct the expression of cell-specific cytokines and chemokines, as well as a myriad of downstream effects [7]. As a key regulator of Th2 responses, IL-33/ST2 triggers the production of Th2 cytokines such as IL-4, IL-5, and IL-13 in Th2 cells [6,46]. In epithelial cells, IL-33 activation of ST2 specifically results in chemokine production [47]. Active signaling by IL-33/ST2 results in activation of immune effector cells [7,43] that leads to recruitment of pro- or anti-tumorigenic cells into the TME in cancer [43]. IL-33 signaling can be attenuated by multiple mechanisms. As indicated above, its pro-inflammatory activities are diminished by its nuclear localization where it is bound to chromatin to repress transcription and is degraded by pro-apoptotic proteases to prevent extracellular release in cells undergoing apoptosis. During activation under conditions of stress, feedback mechanisms attenuate IL-33 signaling to quench the immune responses. Stimulation of ST2L by IL-33 activates focal adhesion kinase (FAK) and glycogen synthase kinase 3β (GSK-3β) [48]. GSK-3β binds to and phosphorylates ST2L; phosphorylated ST2L is rapidly internalized where it is polyubiquitinylated by the E3 ligase FBXL19 and targeted for degradation by the proteasome [48]. IL33/ST2 signaling is also inhibited by a single immunoglobulin domain IL1-R-related molecule (SIGIRR, TIR8) which disrupts the ST2/IL1-RAcP heterodimer [49]. In addition, sST2 negatively regulates IL-33/ST2 signaling by acting as a decoy receptor and sequestering IL-33 to block its interaction with ST2L [11]. Furthermore, IL-33 has been shown to be rapidly inactivated in extracellular space by oxidation of its cysteine residues and formation of disulfide bonds in its IL-1 like cytokine domain [50]. The mechanisms by which IL-33/ST2 signaling is activated and diminished are summarized in Figure 2. In all, IL-33/ST2 signaling can induce inflammatory responses that are cell-dependent and may have opposing effects in allergic or inflammatory conditions or in diseases such as cancer.

## 3. The Role of IL33/ST2 in Tumorigenesis

### 3.1. Breast Cancer

Breast cancer is one of the leading causes of cancer related mortalities among women worldwide. The involvement of IL33/ST2 in cancer was first reported by Jovanovic et al. (2011) [51,52] in breast cancer studies using *ST2^−/−^* mice. Loss of *ST2* in female mice inhibited the growth of 4T1 breast cancer cells and significantly reduced metastasis as compared to wild type mice. Serum levels of IL-17, interferon (IFN)-γ, and TNF-α were elevated, accompanied by increased intra-tumor accumulation of activated natural killer (NK) cells and CD8^+^T cells, suggesting that loss of IL-33 signaling through the ST2 receptor can promote Th1/Th17 polarization of the innate and acquired immune responses [51,52]. Suppression of sST2 was also found to reduce Erb-B2-induced cell motility in breast cancer cells [53]. In subsequent studies [54], repeated administration of IL-33 accelerated tumor growth at the primary orthotopic sites and increased metastasis to the lungs and liver through increased accumulation of immunosuppressive CD11b^+^Gr-1^+^TGF-β1^+^ myeloid derived suppressor cells (MDSCs), IL-13-producing Lin^−^Sca-1^+^ST2^+^ innate lymphoid cells (ILCs), and CD4^+^Foxp3^+^ST2^+^IL-10^+^ regulatory T (T_reg_) cells. IL-33 promoted tumor cell proliferation and angiogenesis in wild type mice but not in *ST2^−/−^* mice. Furthermore, administration of IL-33 to wild type mice significantly reduced the cytotoxic activities of NK cells but not CD8^+^ T cells, although mammary tumor progression was unaffected when CD8^+^ T cells were depleted in vivo [54]. In agreement with these studies, Xiao et al. [55] showed that IL-33 positively regulated the accumulation of MDSCs in the TME by inducing the autocrine secretion of granulocyte-macrophage colony stimulating factor (GM-CSF) to create a positive amplifying loop for recruiting MDSCs into tumor beds. In turn, induced expression of arginase-1 and activation of NF-κB and MAPK signaling by IL-33 in MDSCs augmented their immunosuppressive ability. Loss of *ST2* significantly reduced MDSC accumulation due to defective IL-33 signaling [55].

Studies in *ST2^−/−^* Balb/c mice provided evidence that IL-33/ST2 signaling plays an important role in tumor angiogenesis and necrosis [56]. Orthotopically implanted 4T1 breast cancer cells showed a significant reduction in vascular endothelial growth factor (VEGF) and IL-33 expression as compared to tumors in wild type mice. Consistent with these observations, patient tumors with no necrosis expressed higher levels of IL-33, ST2, and VEGF as compared to necrotic tumors. However, increased microvascular density (MVD) in necrotic tumors positively correlated only with VEGF but not with IL-33 and ST2 expression [56]. Recently, Kim et al. [57] proposed a novel intracellular mechanism by which IL-33 might induce cell proliferation in breast tumor cells based on studies showing that administration of IL-33 increased the phosphorylation of Cancer Osaka Thyroid (COT) protein via a dose- and time-dependent interaction between ST2 and COT. The activated IL-33/ST2/COT cascade induced the MEK-ERK, JNK-c Jun and STAT3 signaling pathways which then promoted the activation of downstream AP-1 and STAT3 transcriptional activities. Clinically, expression of ST2 correlated with elevated COT levels in human breast cancer tissues. Moreover, knockdown of *IL-33*, *ST2* and *COT* significantly reduced the tumorigenicity of breast cancer cells [57].

In patient-based studies, IL-33 expression correlated with breast cancer progression. Patients with ER-positive breast cancer showed increased serum levels of IL-33 and sST2 that correlated with elevated levels of VEGF [58] and the angiogenic factors matrix metalloproteinase-11 (MMP-11) and platelet derived growth factor-c (PDGF-C) [59]. In both cases, these were predictive of poor prognosis [58] and shorter survival time [59]. Serum IL-33 levels were significantly higher in patients with carcinomas as compared to those with benign breast cancer [60]. Histological examination of tissues from these patients showed higher IL-33 expression in breast carcinomas as compared to tumor-adjacent normal tissues [60]. In another study, serum levels and genetic polymorphisms of *IL-12* and *IL-33* were compared among breast cancer patients and healthy cohorts [61]. Although serum IL-12 was significantly lower in patients with stage I breast cancer as compared to the healthy controls, there were no differences between the patients and controls at stages II, III and IV; however, similar to previous reports [54,58,60], serum levels of IL-33 were higher in the breast cancer patients. Serum IL-33 was also higher in patients with stage IV disease resulting in a higher serum ratio of IL-33/IL-22 in patients with stage III and IV diseases as compared to those with stage I and stage II. Genotyping analysis showed that there was no significant change in the frequencies of genotypes and alleles at SNPs rs3212227 and rs1929992 in *IL-12b* and *IL-33* genes, respectively, and that serum levels of IL-12 and IL-33 were not influenced by these genotypes in both patients and healthy controls [61]. Finally, serum IL-33 was used in differential diagnoses of idiopathic granulomatous mastitis (IGM) and breast cancer [62]. IL-33 was significantly higher in IGM patients as compared to breast cancer patients. Although, serum levels of sST2 were significantly lower in IGM patients, there was no significant difference between procalcitonin levels, a clinical marker of inflammation and infection. Thus, serum levels of IL-33 and sST2 could serve as differential diagnostic markers between IGM and breast cancer along with radiological and pathological examinations [62]. In summary, the IL-33/ST2 pathway promotes progression of breast cancer in both pre-clinical and clinical studies mostly through the recruitment of inflammatory cells that enhance tumor growth, invasiveness, and angiogenesis.

### 3.2. Colorectal Cancer

Colorectal cancer (CRC) is the third most frequent cancer and the second leading cause of cancer related deaths in the United States (US) [63]. The role of IL-33/ST2 in CRC carcinogenesis has been extensively studied by many investigators. For an in depth review of the role of IL-33/ST2 in CRC, we refer the readers to a recent review by Akimoto and Takenaga [43]. IL33/ST2 was first implicated in CRC in early studies that showed increased IL-33 expression in CRC patient tissues as compared to non-cancer adjacent tissues, correlating with transition from adenoma to carcinoma progression [64]. Consistently, overexpression of IL-33 in SW620 human colon cancer cells increased tumor growth, migration, and colony formation in vitro and enhanced tumor growth and lung metastasis in vivo, while inhibition of IL-33 had the opposite effect [65]. Subsequently, Maywald et al. used the *Apc^Min/+^* mouse, a genetic model of human familial adenomatous polyposis, to show that IL-33 was localized in tumor epithelial cells while ST2L was expressed in stromal subepithelial myofibroblasts (SEMFs) and mast cells residing within tumor beds [66]. IL-33 induced SEMFs to express components of the extracellular matrix and growth factors that contributed to tumor growth and development [66]. Loss of *IL-33* [66] or *ST2* [67] by genetic knockout inhibited tumor growth, induced apoptosis, and suppressed angiogenesis in *Apc^Min/+^* polyps resulting in decreased tumor burden and size [43,66,68]. Furthermore, loss of *IL-33* reduced infiltration of tumor beds by mast cells and regulatory T (T_reg_) cells, that are both known to be required for polyposis in *Apc^Min/+^* mice [69,70].

**Figure 3:**
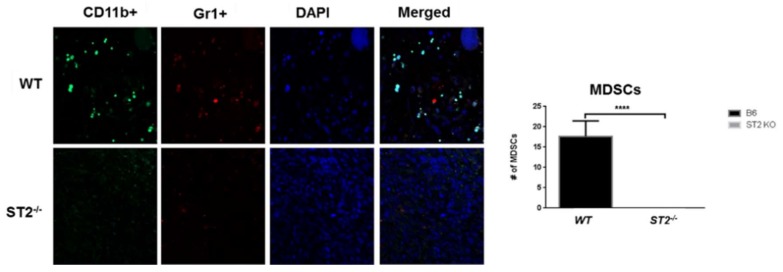
Loss of IL33/ST2 signaling alters the tumor microenvironment. Analysis of tumors derived from murine MC38 colon cancer cells by fluorescent confocal microscopy showed that tumors are highly infiltrated with CD11b^+^Gr1^+^ myeloid derived suppressor cells (MDSCs) in wild type (WT) C57Bl/6 mice (top panel, WT). Loss of host ST2 receptor in *ST2* knockout mice resulted in significant reduction of MDSCs in the TME and inhibition of tumor growth (lower panel, ST2^−/−^). Macrophages, mast cells, and T_regs_ were similarly reduced in *ST2^−/−^* mice indicating that loss of IL33/ST2 signaling alters the composition of the TME (Larsen and Peña, unpublished data; images were taken at 40× magnification on a Zeiss LSM510 META confocal scanning laser microscope, **** indicates a *p* value of < 0.0001).

In an orthotopic model of CRC liver metastasis, overexpression of IL-33 in mouse CT26 and MC-38 CRC cells enhanced tumor take, tumor growth and liver metastasis when injected into the cecum of syngeneic host mice [68]. Tumor-rather than host-derived IL-33 directed the recruitment of CD11b^+^ Gr1^+^ MDSCs and CD11b^+^ F4/80^+^ macrophages [68] as well as CD117^+^ FcRεγ^+^ mast cells (Pena et al., unpublished data and [71]) into the tumor beds to remodel the TME and promote growth, invasion, angiogenesis, and metastasis. Recruitment of MDSCs and macrophages promoted the release of VEGF and S100A8/9 molecules, respectively, to enhance angiogenesis and invasiveness of tumor cells [68]. Loss of host ST2L (Larsen and Pena, unpublished data) but not host IL-33 [68] inhibited the recruitment of these cells into the TME suggesting that IL-33/ST2 signaling is an important determinant of the immune composition of the TME (Figure 3). 

Elegant studies by Akimoto et al. showed that expression of sST2 was inversely associated with malignant growth of CRC and was downregulated in highly metastatic cells compared with low metastatic cells [72]. Knockdown of sST2 in low-metastatic cells enhanced tumor growth, metastasis and angiogenesis, while its overexpression in high-metastatic cells suppressed these processes [72]. Overexpression of circulating levels of an sST2-Fc protein fusion in tumor bearing mice reduced growth and metastasis of highly metastatic CRC cells by suppressing angiogenesis, Th1- and Th2-immune responses, macrophage infiltration and M2a macrophage polarization [72]. Thus, sST2 acted as a negative regulator of CRC malignancy by remodeling the TME. More recent studies showed that IL-33 is expressed by vascular endothelial cells and tumor cells in human CRC [73]. Administration and overexpression of IL-33 induced tumor sphere formation and protected cells from chemotherapy-induced apoptosis. These studies suggest that IL-33/ST2 signaling induced CRC stemness by activating core stem cell genes such as *NANOG, NOTCH3,* and *OCT3/4*, and by inducing the phosphorylation of c-Jun N terminal kinase (JNK) to activate c-Jun and enhance its binding to the promoters of these genes [73]. IL-33 induced the recruitment of macrophages into the TME where they produced prostaglandin E2 that further supported CRC stemness and tumor growth. Thus, IL-33 targeted both tumor cells and macrophages to confer a novel immune-mediated mechanism to promote CRC malignancy. Tumor expression of IL-33 was associated with poor survival and can potentially be targeted to treat metastatic CRC [73].

In other studies, the role of epithelial IL-33 in intestinal tumor development was examined in *Apc^Min/+^* mice transgenically expressing IL-33 in intestinal epithelial cells (V33 *Apc^Min/+^*) [74]. These mice had increased intestinal tumor burden as compared to littermate *Apc^Min/+^*. Consistently, *Apc^Min/+^* mice lacking the ST2 receptor had reduced polyp burden. Mechanistically, overexpression of IL-33 promoted the expansion of ST2^+^ regulatory T cells, increased Th2 cytokine production, and induced alternative activation of macrophages in the gut. IL-33 also promoted significant changes in the expression of antimicrobial peptides, and antibiotic treatment of V33 *Apc^Min/+^* mice abrogated the tumor promoting-effects of IL-33 in the colon [74]. Thus, elevated epithelial IL-33 signaling increased tumor development in the *Apc^Min/+^* mice [74].

In contrast to the tumor promoting effects of IL-33/ST2 signaling described above, shRNA mediated knockdown of *ST2* in murine CT26 cells enhanced tumor growth in Balb/c mice suggesting an anti-tumorigenic role in CRC [47]. This was associated with decreased macrophage infiltration, likely a consequence of reduced induction of *CCL2* by IL-33 in the absence of IL-33/ST2 signaling. ST2L expression was found to be reduced in human colon tumors as compared with adjacent non-tumor regions and inversely correlated with higher tumor grade [47]. In a number of studies, immuno-histochemical staining of tissue samples from patients and mouse models as well as measurement of mRNA expression of IL-33 and ST2 in tumor samples have collectively reported higher levels in adenomas and early stage CRC [64,65,66,68] as compared to higher-grade and more advanced or late stage tumors, suggesting that IL-33/ST2 signaling may play an anti-tumorigenic role in later stages of CRC. In elegant studies using a sporadic model of non-inflammatory CRC, loss of *ST2* enhanced tumor development while administration of IL-33 reduced growth of CRC allografts [75]. Using reciprocal bone marrow chimeras, simultaneous loss of IL-33 signaling in both radioresistant non-hematopoietic and radiosensitive hematopoietic compartments resulted in increased tumor burden. Stimulation of ST2 by IL-33 in the radioresistant mesenchymal compartment induced the expression of NF-κB target genes which further led to the recruitment of regulatory T cells and suppression of IFN-γ mediated gene expression. Decrease in IFN-γ was associated with more aggressive CRC in human patients indicating that loss of IL-33 signaling impaired a potent IFN-γ mediated anti-tumor immune response [75].

In summary, opposing roles for IL-33/ST2 signaling has been reported in CRC development and malignancy. Both tumor and stromal cells have been shown to express IL-33, ST2L, and sST2 thus IL-33/ST2 signaling can potentially function in an autocrine or paracrine fashion to stimulate tumor cells as well as stromal cells in the TME. Regardless of the source of IL-33, whether tumor-derived or stromal, the overall impact of IL-33/ST2 signaling lies in its ability to direct the remodeling of the TME. Under pro-tumorigenic conditions, IL-33 directed the recruitment of tumor promoting inflammatory immune cells such as MDSCs, mast cells, macrophages, and T_regs_. In contrast, under anti-tumorigenic conditions, IL-33 directed the recruitment of NK cells and CD8^+^T cells, and increased production of IFN-γ that favor eradication of tumor cells [76,77]. Further studies need to be conducted to resolve the controversial role of IL-33/ST2 in CRC progression to realize its potential as a therapeutic target or as adjuvant therapy to enhance the efficacy of antitumor immunotherapies.

### 3.3. Gastric Cancer

The development of intestinal-type gastric cancer is typically preceded by the emergence of metaplastic cell lineages in the gastric mucosa [78]. Specifically, intestinal metaplasia and spasmolytic polypeptide expressing metaplasia (SPEM) have been implicated with the pathological progression to intestinal-type gastric cancer. SPEM development occurs as a physiological response to mucosal injury which results in the recruitment of mucus secreting cells that repair the damage by adding a protective barrier to the epithelium. IL-33 is expressed in a subset of surface mucous foveolar epithelial cells and is released upon mucosal damage [79]. It is thought that type II innate lymphoid cells then respond to IL-33 to produce cytokines including IL-13. Downstream signaling from IL-13 induces SPEM formation and the alternative activation of macrophages [79]. These M2a-polarized macrophages then secrete more IL-33 to create a self-sustaining signaling network that promotes progression of SPEM to a more proliferative metaplasia. In this case, a mechanism for physiological repair of an acute injury can progress into a self-sustaining cascade that becomes a risk for gastric cancer development in the presence of persistent injury and chronic inflammation mediated by IL-33 and IL-13 [78].

### 3.4. Hepatocellular Carcinoma

Hepatocellular carcinoma (HCC) is the most common primary malignant liver tumor that typically arises during liver cirrhosis (LC) and has a poor prognosis [80]. Serum levels of IL-33 were elevated in patients with HCC [81,82]. This correlated with increased expression of IFN-α, IFN-γ, and IL-33 cytokines that were further increased in patients with metastatic HCC as compared to patients with non-metastatic HCC [82]. Expression of IL-33 in resected HCC tissues was analyzed by immunohistochemistry and flow cytometry to assess its effect on patient survival and on the immunological and molecular TME [81]. The data showed that localized infiltration of IL-33^+^ cells within the tumor and infiltrative margins was associated with increased patient survival, with more cells localized in the near and distant stroma as compared to tumor regions. Flow cytometry and double immunofluorescence analyses showed that IL-33 was produced by CD8^+^ cells, specifically, the CD45^+^CD8^+^CD62L^–^KLRG1^+^ effector memory cells. These cells express the ST2 receptor in the fraction that was positive for the CD107a marker which identifies cells that have been cytotoxically active. This study showed for the first time that the CD8^+^T cells mediating the cytotoxic effects in the TME were an important source of IL-33 [81]. This further led to the recruitment of cytotoxic NK T cells producing IL-13. Increased infiltration of tumor and distant stromal regions correlated with increased patient survival [81]. Based on the infiltration of the IL-33^+^ and CD8^+^ cells, an HCC immune score was developed to identify high- and low-risk patients. Microarray analysis confirmed a distinct gene expression pattern between these two groups of patients. The HCC immune scores provided a risk stratification mechanism for HCC patients that was useful in clinical evaluation [81].

In another case-control patient based study, serum levels of IL-33 and sST2 were measured in patients with HCC, LC, and in healthy controls (HC) [80]. In contrast to the study above, there were no significant differences in IL-33 serum levels in patients with HCC as compared to LC and HC controls. There was no correlation between IL-33 levels and overall survival (OS), liver function parameters, scores for End-Stage Liver Disease (MELD) and staging for HCC [CLIP (cancer of the liver Italian Program) score]. On the other hand, sST2 levels were significantly elevated in LC and HCC patients as compared to HCs and there was significant correlation with OS, liver parameters, MELD and CLIP scores. Thus, serum sST2 might be used as a new prognostic marker in HCC [80].

In a recent study, genetic variants in the IL-33/ST2 pathway were analyzed to determine their association with susceptibility to HCC in a Chinese population [83]. Genotyping of the *IL-33* rs7025417 and ST2 rs3821204 alleles showed that the *ST2* rs3821204 CC genotype was associated with a significantly increased risk of HCC that was more evident in smokers and drinkers. Higher plasma levels of ST2 mRNA and protein suggested an increased risk due to enhanced ST2 production at the transcriptional and translational levels [83].

### 3.5. Hepatobiliary Cancers

Hepatobiliary inflammation and fibrosis are risk factors in humans for cholangiocarcinoma (CCA), a lethal hepatobiliary cancer originating from the biliary apparatus [84]. In a mouse model of CCA, administration of IL-33 in combination with ectopic expression of constitutively active AKT (myr-AKT) and Yes-activated protein (YAP) oncogenes induced tumor development in 72% of mice as compared to 20% in the absence of IL-33. Like human CCA, the tumors in mice expressed PanCK and SOX9 but not HepPar1, a marker of hepatocellular carcinoma. In addition, murine tumors had abundant infiltration of myofibroblasts expressing α-smooth muscle actin (α-SMA), elevated serum levels of CA 19-9 antigen, and mRNA expression profiles that mimic human CCA. Expression of ST2 was also higher in murine tumors and human CCA samples. Mechanistically, IL-33 facilitated tumor development in vivo through the up regulation of IL-6. Loss of IL-6 in *IL-6^−/−^* male mice significantly attenuated CCA development. Systemic administration of IL-6 in the CCA murine model induced tumor development at a similar rate to IL-33 [84]. This model highlights the role of inflammatory cytokines, specifically IL-33 and IL-6 in CCA carcinogenesis.

Injury to the biliary epithelium can trigger inflammation and fibrosis that can result in severe liver diseases that may progress to malignancy [85]. Although type 1 immune responses have been linked to the pathogenesis of biliary injury, Th2 promoting cytokines were elevated in a subset of patients with biliary artesia, the most common childhood cholangiopathy. Elevated IL-33 levels in sera of patients with biliary artesia and in livers and bile ducts of mice with experimental artesia was proposed to be the underlying instigator [85]. Exogenous IL-33 served as a potent mitogen of epithelial cells in the biliary region and induced a dramatic enlargement of hepatic bile ducts. The increased proliferation in response to IL-33 was mediated by the accumulation of type-2 innate lymphoid cells (ILC2s) that released high levels of IL-13 that in turn promoted cholangiocyte hyperplasia. Induction of the IL-33/ILC2/IL-13 circuit promoted epithelial repair, however, induction of this circuit in mice with constitutively activated oncogenic AKT and YAP in bile ducts induced cholangiocarcinoma with liver metastases [85]. Thus, disruption of IL-33/ILC2/IL-13 circuit may block the progression of biliary carcinogenesis [85]. These studies established the role of IL-33 as a biliary mitogen that promotes inflammation and fibrosis.

### 3.6. Pancreatic Cancer

Human pancreatic cancer is one of the most deadly malignancies with a five year survival rate of only 8.2% [86]. Despite the advances in surgery and oncology, the survival rate remains dismal for pancreatic cancer patients. Inflammation of the pancreas plays a critical role in the development of pancreatic cancer and is driven by inflammatory pathways. The precise role of IL-33 in pancreatic inflammation and cancer remains to be elucidated. However, studies in patients and mouse models of pancreatic inflammation that precede malignancy have implicated IL-33/ST2 signaling. IL-33 is expressed in the nucleus of pancreatic stellate cells (PSCs) [87] that when activated, play a pivotal role in pancreatic fibrosis that is found in chronic pancreatitis and pancreatic cancer. Expression of nuclear IL-33 in activated PSCs was increased by inflammatory factors such as IL-1β, TNF-α, PDGF-BB, and IFN-γ, but not TGF-β1 [87]. Nuclear expression of IL-33 was also observed in activated human and rat PSCs and in human tissues of chronic pancreatitis and pancreatic cancer. In studies using inhibitors of NF-κB (Bay11–7082), the ERK pathway (U0126), p38 MAPK signaling (SB203580), the JNK pathway (SP600125), and the phosphatidylinositol 3-kinase-Akt pathway (wortmannin), it was shown that IL-1β induced the expression of IL-33 through NF-κB or ERK pathways and partially through the p38 MAPK pathway while PDGF-BB does the same mainly through the ERK pathway [87]. PSCs express sST2, ST2L and IL-1RAcP, however, expression of ST2L was significantly lower than that in human vascular endothelial cells (HUVECs). Interestingly, recombinant IL-33 did not induce the proliferation and migration of PSCs but knockdown of IL-33 inhibited PDGF-BB induced proliferation [87].

Studies in the highly metastatic human pancreatic adenocarcinoma cell line Colo357 showed that IL-33 is a critical mediator of inflammation-associated pancreatic tumorigenesis and modulates its own receptor levels [88]. Expression of IL-33 mRNA was induced by the pro-inflammatory cytokines IL-1 and IL-3 but was downregulated by IL-12. Interestingly, IL-33 and IL-1 showed counter-regulatory effects on the expression of sST2 and ST2L mRNA. Moreover, IL-33 and IL-1 synergistically up regulated the expression of IL-6 but only IL-33 up regulated IL-8 expression independent of IL-1. The induction of these inflammatory cytokines in the presence of IL-33 and IL-1 was mediated through NF-κB activation in Colo357 cells [88].

### 3.7. Lung Cancer

Lung cancer is the leading cause of cancer-related deaths in the United States and worldwide. The Centers for Disease Control (CDC) reported that more people in the U.S. die from lung cancer than any other type of cancer [86]. While lung cancer rates have dropped in the U.S. within the past decade, mostly due to successful education on the direct link between cigarette smoking and lung cancer, understanding the underlying biological mechanisms that drive its progression is imperative for successful prevention or management of the disease. The involvement of IL-33 in lung cancer was first studied by Naumnik et al. in 2012 [89] to assess its potential as a diagnostic marker for advanced stages of lung cancer. Sera and bronchoalveolar lavage fluid (BALF) taken prior to chemotherapy from patients diagnosed with non-small cell lung cancer (NSCLC) were analyzed by enzyme linked immunosorbent assay (ELISA) to assess circulating levels of IL-27, IL-29, IL-31, and IL-33. Among the four groups of patients with NSCLC, sarcoidosis (BBS), hypersensitivity pneumonitis (HO) and healthy controls that were studied, serum levels of IL-27, IL-31, and IL-33 did not differ, while BALF IL-33 levels did not change before or after chemotherapy [89] and therefore, were not predictive for lung cancer. In contrast, similar patient-based studies suggested that serum IL-33 may be a useful diagnostic biomarker in lung cancer. In this study, IL-33 was elevated in patients with NSCLC when compared to healthy volunteers (HV) and patients with benign lung diseases (BLD) [90]. In particular, baseline IL-33 levels were found to be an independent prognostic factor in a subgroup of patients who received active treatment for locally-advanced or metastatic disease [90]. Consistent with a pro-tumorigenic role, IL-33 and ST2 expression in patient tumor tissues were found to correlate with tumor progression in NSCLC patients. Overexpression of IL-33 by transfection into NSCLC cells isolated from patients [91] and treatment of human lung A549 cells with IL-33 [92] enhanced tumor outgrowth, migration, invasion, and metastasis in an ST2-dependent manner. Genetic knockdown of IL-33 with shRNA or blockade of ST2 limited NSCLC progression [91]. These studies identified the membrane-bound glucose transporter 1 (GLUT1) as a novel target of IL-33/ST2 signaling resulting in enhanced glucose uptake and glycolysis in NSCLC cells. Targeting GLUT1 abrogated IL-33 induced growth and metastasis of NSCLC [91]. Later studies showed that IL-33 blockade inhibited tumor growth by abrogating the polarization of M2 tumor-associated macrophages (TAMS) and reducing accumulation of T_regs_ in the TME thereby shaping functional immune surveillance [93]. Finally, a frequent post-operative complication in NSCLC patients is pulmonary infection with gram-negative bacteria [94]. Bacterial infection correlated with advanced disease stage and shorter interval to disease recurrence in patients. Infection of patient-derived NSCLC cells with gram-negative bacteria enhanced tumor proliferation, invasion, and metastasis by activation of *TLR4* in NSCLC cells resulting in increased IL-33 expression though a MyD88-dependent pathway [94].

An anti-tumorigenic role for IL-33 in lung cancer was proposed in studies showing that male patients who had a history of smoking had significantly lower plasma IL-33 levels as compared to healthy controls and that IL-33 expression was inversely associated with progression of lung cancer [95]. Activation of epithelial and endothelial cells during the early stages of lung cancer was thought to trigger the release of IL-33. However, a reduction in lung volume in the later stage of the disease may cause a decrease in IL-33 levels [95]. In addition, reduced lung volume due to surgical removal of the tumor may also decrease IL-33 levels by diminishing bronchial and vascular endothelial volumes [95]. Similarly, Akimoto et al. [96] showed that ST2 was significantly downregulated in human lung cancer as compared to normal lung tissues and cells. IL-33 expression was inversely correlated with progressive stages of human lung cancers [96,97] and lower levels were associated with poor prognosis of pulmonary carcinoma [97]. Consistent with this finding, low metastatic but not highly metastatic cells derived from LLC expressed high levels of ST2 [96]. Although the low metastatic cells expressed low levels of IL-33, tumors derived from these cells abundantly expressed IL-33 in vivo as compared to tumors from high metastatic cells. IL-33 enhanced the death of ST2L-positive low-metastatic cells but not ST2L-negative high metastatic cells in glucose-depleted, glutamine-depleted hypoxic conditions through the activation of p38 MAPK and mammalian target of rapamycin (mTOR). Cell death occurred by oncosis, and IL-33 mediated the selection of ST2L-positive, oncosis-resistant high-metastatic cells by establishing conditions that simulate the TMN [96]. In other studies using mouse models of lung metastasis, transgenic expression of *IL-33* in Lewis Lung carcinoma (LLC) and B16 melanoma cells attenuated tumor metastasis. Mechanistically, IL-33 stimulated NF-κB signaling to promote proliferation, activation, and enhanced recruitment and cytotoxicity of CD8^+^ T cells and NK cells [76] into tumors to block pulmonary metastasis. Depletion of CD8^+^ T cells and NK cells abolished the inhibition of metastasis by IL-33 [76]. In subsequent studies, Gao et al. [77] showed that over-expression of IL-33 in B16 melanoma and 4T1 breast cancer cells inhibited tumor growth and metastasis by increasing the numbers and IFN-γ production of CD8^+^ T and NK cells in tumor tissues, creating a TME that favored the eradication of tumors [77]. More recent studies showed that IL-33 can regulate cytokines to mobilize Type 2 innate lymphoid cells (ILC2) from the lung and other tissues into tumor beds where they orchestrate tumor immune-surveillance in cooperation with dendritic cells to promote cytolytic T cell responses, thereby enhancing anti-cancer immunity and controlling metastasis [98].

Although extensive research has been carried out to elucidate the functions of IL-33 in lung cancer, there are conflicting data as to whether IL-33 is a pro- or anti-tumorigenic cytokine. These discrepancies suggest that the role of IL-33 in lung cancer may be context dependent and therefore additional studies evaluating the signaling mechanisms by which IL-33 functions are necessary. 

### 3.8. Prostate and Kidney Cancer

Studies in prostate and kidney renal clear cell carcinoma revealed a novel mechanism for metastatic immune escape through the loss of expression of IL-33 [99]. Using data from the Gene Expression Atlas created by the European Biostatistics Institute (Gene Expression Atlas; Available online: https://www.ebi.ac.uk/gxa/home; access date: 30 January 2013), IL-33 expression was found to be reduced in human metastatic prostate cancer as compared to benign and primary prostate tumors. IL-33 expression positively correlated with expression of major histocompatibility complex 1 (MHC-I) [99]. RNA sequencing data collected from resected human prostate tumors further established that similar to *IL-33*, expression of human leukocyte antigens (HLA) *HLA-A*, *HLA-B*, *HLA-C* in high risk neo-hormone treated (NHT) and treated metastatic castration-resistant prostate cancer (CRPC) were also reduced [99]. Similar observations also held true for *TAP1*, *CD4*, and *CD8* genes. These data suggested that during metastatic re-programming, lower levels of IL-33 and MHC-I may contribute towards immune escape of prostate cancer. Overexpression of IL-33 in metastatic A9 cells induced the MHC-I which in turn significantly reduced the metastatic ability of these cells. Moreover, accumulation of CD8^+^T cells in tumor beds of A9+IL-33 derived tumors were higher as compared to A9+vector and TC1+vector derived tumors. On the other hand, the number of suppressor T cells (FoxP3^+^) was higher in the tumor beds of A9+vector as compared to A9+IL-33 [99]. Thus, IL-33 over expression in metastatic tumors may serve as a marker of an anti-tumor response against it. Interestingly, prostate cancer patients with higher IL-33 expression had a prolonged recurrence time of 97 months as compared to 56.7 months in patients with lower IL-33. Similarly, lower levels of IL-33 were associated with shorter survival time in patients with kidney renal clear cell carcinoma as compared to patients with higher IL-33 expression. Thus, IL-33 may potentially be used as a generalized therapy for metastatic cancers to restore immune-surveillance and counter immune escape mechanisms [99].

The effect of IL-33 deficiency on acute kidney injury (AKI) and cancer growth was studied in a four-week model of cisplatin-induced AKI in mice with cancer [100]. An increase in kidney levels of IL-33 was found to precede AKI and tubular injury, suggesting that IL-33 may play a causative role. However, there were no differences observed between wild type and IL-33 deficient mice with respect to increases in molecular indicators of kidney damage. Although kidney expression of the pro-inflammatory cytokines CXCL1 and TNF-α increased in *IL-33* deficient mice, surprisingly, tumor weight, volume, and growth were significantly decreased and the effect of cis-platin on tumors was greatly enhanced. Serum IL-33 also increased in cis-platin induced AKI mice as well as in AKI patients post-surgery as compared to non-AKI patients. Overall, the study indicated that IL-33 does not protect against AKI in a clinically relevant model and may not be a useful therapeutic target. However, serum IL-33 may be a relevant biomarker for AKI [100].

### 3.9. Ovarian Cancer

Ovarian cancer is a heterogeneous type of malignancy with a high rate of mortality among women worldwide. Epithelial ovarian cancer (EOC) is the most lethal form and accounts for 80–90% of all cases [63]. To examine the role of IL-33/ST2 in the growth and metastasis of EOC, expression levels of IL-33 and ST2 were determined in patient tumor tissues and in the human EOC cell lines (HO8910, CAOV3 and SKOV3-DDP) [101]. IL-33 and ST2 were both aberrantly expressed in EOC tissues as compared to normal ovary tissue and benign ovarian tumors, with much higher expression in tumors from metastatic sites. IL-33 and ST2 levels in EOC metastasis did not correlate with patient age or pathological characteristics. However, it was positively correlated with Ki-67 expression and reduced patient survival time. Knock down of IL-33 with siRNA in EOC cell lines significantly reduced their migration and invasion potentials, while overexpression of full length hIL-33 significantly induced their migratory and invasive properties and their proliferation rates, both of which were inhibited by the decoy receptor sST2 [101]. Mechanistically, IL-33 mediated these effects through phosphorylation of ERK and JNK; treatment with ERK or JNK inhibitors either fully reversed or reversed only IL-33 mediated effects, respectively. In all, these studies indicated that IL33/ST2 is predictive of poor prognosis in EOC patients, promotes ovarian cancer growth and metastasis through the ERK and JNK signaling pathways, and might be potential prognostic markers or therapeutic targets [101].

### 3.10. Uterine Cancer

Uterine leiomyomas, also called uterine fibroids or myomas, are benign smooth muscle tumors of the myometrium that may cause excessive uterine bleeding, pelvic pain, recurrent miscarriages, and infertility [102]. These are the most common benign tumors in women of reproductive age and have been diagnosed in approximately 25% of this cohort. Increased smooth muscle cell proliferation and excessive extracellular matrix (ECM) deposition are the two essential features of all leiomyoma tumors. In early studies, serum IL-33 was found to be abnormally elevated in women with endometriosis, particularly those with deeply infiltrating endometriosis (DIE) [103]. This correlated with the intensity of preoperative painful symptoms and the extent of severity of DIE. To assess the role of IL-33 in uterine leiomyoma tumor burden, serum levels were measured in women with uterine leiomyoma and leiomyoma-free controls after complete surgical exploration of the abdominopelvic cavity [102]. Women with benign ovarian cysts, paratubal cysts or tubal defects without any evidence of uterine leiomyoma were considered as controls. IL-33 was detected in sera of ~50% leiomyoma patients and ~20% of control patients. However, serum levels of IL-33 were significantly higher in leiomyoma patients as compared to the controls. Serum concentrations of IL-33 were positively correlated with the pathology of the disease such as fibroid weight and the size and number of fibroids. Although this study may have a selection bias due to the inclusion of only surgical patients, wherein control patients who have also undergone surgeries for benign gynecological conditions might also exhibit elevated IL-33 levels, it demonstrated for the first time an association between elevated serum IL-33 and the existence of uterine leiomyoma. Further studies are needed to better understand the mechanisms by which IL-33 may enhance tumor burden in these patients [102].

### 3.11. Endometrial Cancer

Endometrial cancer is the second most common cancer in females which occurs most frequently during perimenopause and menopause, between the ages of 50 and 65, worldwide. Recent studies showed that IL-31 and IL-33 protein levels were significantly elevated in sera from patients with endometrial cancer as compared to healthy controls [104]. These levels positively correlated with clinical characteristics such as tumor stage, depth of invasion, existence of node metastases, and distant metastases. Interestingly, the sensitivity and specificity of IL-31 and IL-33 expression and detection were higher than the typical endometrial cancer biomarkers CAE, CA-125, and CA19-9. Thus, in this very first study linking endometrial cancer with IL-31 and IL-33, these interleukins could serve as better biomarkers for endometrial cancer diagnosis and prognosis [104].

### 3.12. Cervical Cancer

Infection of high risk human papillomavirus and a defect in anti-viral immunity are closely related to the development of cervical intraepithelial neoplasia (CIN) and carcinogenesis. The involvement of IL-33 in disease progression in human papilloma virus (HPV)-positive patients was examined due to its role in driving protective anti-viral immunity and to determine its association with IFN-γ that has been shown to induce IL-33 expression in epithelial cells [105]. IL-33 was expressed at various stages of the disease in HPV-infected patients. However, there was no statistically significant difference in IL-33 levels in cervical lavage and sera of CIN patients among various stages of disease progression. Protein and mRNA levels of IL-33 in cervical tissues were significantly lower in severe CIN patients as compared to mild or no CIN patients. Similarly, IFN-γ mRNA levels were significantly lower in severe CIN patients. In vitro studies showed that IFN-γ upregulated IL-33 expression in human epidermal keratinocytes (NHEKs) in a dose-dependent manner [105]. However, no further reduction of IL-33 protein and mRNA levels were observed during progression from severe CIN to cervical cancer (CA) even though IFN-γ mRNA levels were higher in CA than in severe CIN. These results suggested that reduced IL-33 expression in severe lesions in precancerous cervical tissues might be a consequence of diminished local IFN-γ levels.

### 3.13. Head and Neck Cancer

There is not a lot of research on the role of IL-33 in head and neck cancer, but an early study by Chen et al. [106] suggested that elevated IL-33 expression was associated with poor prognosis in terms of nodal metastasis-free survival. An organotypic culture was utilized to investigate the involvement of carcinoma-associated fibroblasts (CAFs) in promoting aggressive behavior of head and neck squamous cell carcinoma (HNSCC). Microarray analyses showed that IL-33 was abundantly expressed in CAFs and identified IL-33 as a critical mediator in CAF-induced invasiveness. Inhibition of IL-33 diminished the CAF-induced aggressive behavior of HNPCC while administration of IL-33 promoted cancer cell invasion and migration by inducing epithelial to mesenchymal trans differentiation and increased IL-33 expression in cancer cells [106]. In patient tissues, IL-33 expression in CAFs correlated with IL-33 expression in cancer cells. Patients exhibiting a low invasion pattern grading score (IPGS) were associated with low or no expression of IL-33, whereas patients exhibiting a high IPGS displayed over-expression of IL-33 in both CAFs and cancer cells [106]. These findings suggested that IL-33 is a potential prognostic biomarker that should be considered in therapeutic strategies for the treatment of patients with HNSCC [106].

### 3.14. Brain Tumors and Gliomas

Glioblastoma is the most common primary brain tumor and the most aggressive among all gliomas [107]. Analysis of human glioma tissue arrays by immunohistochemistry showed that IL-33 was expressed heterogeneously in tumor tissue but was undetectable in normal brain tissue. There were no differences in IL-33 expression in different tumor grades but IL-33 protein level was associated with poor survival in patients with recurrent glioblastomas [107]. Analysis of mRNA expression of IL-33, ST2, and IL1-RAcP receptor accessory proteins using the Cancer Genome Atlas database showed that their expression levels were varied; however, IL-33 and IL1-RAcP mRNA levels correlated with tumor grade.

In a rat model of glioma, global gene expression in two rat C6 glioma cell clones with different capacities for tumorigenesis, C6-1 and C6-2, was determined by microarray analysis to identify genes associated with enhanced tumorigenic properties [108]. Among the upregulated genes that were related to proliferation, the highly tumorigenic C6 cells expressed elevated levels of cytokines and chemokines, and among these were IL-33 and its receptor ST2. Blockade of IL-33 or ST2 attenuated cell growth and colony formation as well as cell migration and expression of glioma-associated growth factors and chemokines. In all, IL-33 mediated the development of a microenvironment that facilitated microglia/macrophage infiltration to promote glioma growth in the brain [108].

### 3.15. Gingivitis and Salivary Tumors

The role of IL-33 in inflammation driven disorders has recently been studied extensively in gingivitis. Tada et al. (2016) [109] investigated the role of *Porphyromonas gingivalis*, a periodontal pathogen in the over-expression of IL-33 in human gingival epithelial cells. *P. gingivalis* was identified as the major pathogen that is associated with chronic periodontitis, and its bacterial components induced inflammatory responses [109]. IL-33 expression was assessed by immunohistochemistry in gingival tissues from patients with chronic periodontitis. Expression of IL-33 increased in patients with chronic periodontitis as compared to healthy patients [109]. Furthermore, the elevated expression of IL-33 induced by *P. gingivalis* was mediated by protease-activated receptor-2 (PAR-2) through gingipain (protease)-dependent activation as well as the phospholipase C, p38, and NF-κB signaling pathways in human gingival epithelial cells. This study provided evidence that *P. gingivalis*-induced IL-33 expression was associated with the pathogenesis of periodontitis [109].

Using polyomavirus (PyV)-infected mice as a model of tumorigenesis, Mishra et al. [110] showed that cells from (PyV)-induced salivary gland tumors were efficiently killed by NK cells in vitro in a process that is dependent on interaction between NKG2D (effector cell) and RAE-1 (target cell) [110]. However, in T cell-deficient mice, NK cells only delayed but did not prevent the development of PyV-induced tumors. This was attributed to increased infiltration by CD11b^+^/F4/80^+^ macrophages that induced tumor cells to produce high levels of pro-inflammatory cytokines such as IL-1α, IL-1β, IL-33 and TNF that can each down-regulate RAE-1 expression in tumor cells resulting in resistance to NK cell-mediated killing [110]. This mechanism of immune evasion that is mediated by tumor infiltrating cells appears to be a widespread phenomenon in studies of IL-33 in cancer immunotherapy.

In a study on squamous cell carcinoma of the tongue, patient tumor tissues were analyzed by immunohistochemistry for levels of IL-33 and ST2. Patients with high IL-33 expression had significantly worse prognosis as compared to those with low IL-33 [111]. ST2 expression was also associated with poor prognosis and correlated with IL-33. The impact of IL-33 on the TME was assessed by measuring mast cell density (MCD) and microvessel density (MVD) in the tumor stroma. The data showed that MVD in the tumor stroma was significantly higher in patients with elevated IL-33 expression, suggesting that the IL-33/ST2 axis can promote malignancy and tumor aggressiveness by remodeling the TME [111]. The authors suggest that assessment of IL-33 and ST2 by immunohistochemistry might be useful for identifying patients that may be of high risk for poor prognosis.

In contrast to the study above, immunohistochemical analysis of nuclear IL-33 expression in patients with benign and malignant salivary gland tumors [112] showed that IL-33 was most highly expressed in benign tumors. It was also highly expressed in Warthin’s tumors which are benign cystic tumors also known as papillary cystadenoma lymphomatosum. These tumors showed a strong and consistent IL-33 expression in the basally oriented cells of the epithelial bilayer [112]. On the other hand, in malignant salivary gland tumors, nuclear IL-33 expression was limited to epithelial-myoepithelial, acinic, and oncolytic carcinomas. Increased expression of IL-33 in these particular malignant cells significantly correlated with prolonged survival, lack of metastasis, and favorable histological parameters as compared to IL-33-negative tumors [112]. Poorly differentiated malignant salivary gland tumors that were devoid of myoepithelial cells, such as salivary duct carcinoma, did not express IL-33 which was indicative of poor prognosis [112]. Under these circumstances, IL-33 was deemed as a novel prognostic marker for malignant salivary gland tumors with potential use in clinical diagnostics.

### 3.16. Skin Cancer and Inflammatory Diseases

Early studies on the role of IL-33 in skin cancer concluded that inflammatory doses of UVB significantly induced IL-33 expression within the epidermal and dermal layers of both mice and humans [113]. UV irradiation from sunlight has wide-ranging immunosuppressive capabilities that can be detrimental or protective. It can initiate skin carcinogenesis or protect against a host of inflammatory conditions through various immunosuppressive mechanisms or mediate the production of Vitamin D and antimicrobial peptides [113]. Exposure of murine and primary human cell lines to ssUV and oxidized lipid platelet activating factor in vitro induced IL-33 expression in both epidermal keratinocytes and dermal fibroblasts [113]. IL-33 levels were elevated in the nucleus and cytoplasm of exposed cells, with more prominent nuclear staining in UV-exposed fibroblasts. These results suggested that IL-33 may act as an important danger signal that is produced in response to inflammation inducing radiation. This study further demonstrated that administration of IL-33 to mice in vivo suppressed the induction of Th1-mediated contact hypersensitivity responses [113]. Correspondingly, UV-induced squamous cell carcinomas that evade the immune system were found to express significantly higher levels of IL-33. Dermal mast cells and skin-infiltrating neutrophils were found to closely associate with UV-induced IL-33-expressing fibroblasts [113].

In contrast, studies on atopic dermatitis (AD), an inflammatory skin disorder showed that the production of IL-33 was driven by inflammation signaling mechanisms [114]. In a study by Bergot et al. (2015) [114], the human papilloma virus (HPV) 16 E7 oncoprotein under the control of the keratin promoter was transgenically expressed in wild type and *ST2^−/−^* mice to examine its impact on skin pathology. The results showed that IL-33 was not required for the development of E7-driven skin pathology [114]. Interestingly, given that AD is typically a Th2 driven inflammatory disorder, it was thought that mast cell and T cell infiltration may contribute to E7 pathology. However, through the use of E7. *Kit^W−sh/W−sh^* and E7.Rag1^−/−^ mice, it was found that the skin lesions developed independently of T cells and mast cells [114]. Thus, conflicting data can be seen with regards to the function of IL-33 on inflammation-driven skin disorders and dermal inflammation as reported in these studies [113,114]. These differences may be attributed to many factors including, but not limited to, the use of different mouse models, different cell lines, or may have arisen because the investigators were studying two different skin conditions. It is difficult to decipher the function of IL-33 given the complexities of each individual disease.

In other studies, the splice variants of IL-33 in normal and transformed skin cells were determined. These included normal human dermal fibroblasts (NHDF), fibroblasts from dermatofibrosarcoma protuberans (DFSP), neonatal foreskin, normal human epidermal keratinocytes (NHEKs), the melanoma cell lines MM-AN, MM-BP, MM-LH, MM-RU, and RPM-MC and a human squamous cell carcinoma cell line (DJM). NHDF cells were found to express lower levels of IL-33 as compared to its transformed variant, the DFSP cells [115]. Transfection of different splice variants resulted in either nuclear (full-length IL-33, or variants lacking exon 4 and/or 5) or cytoplasmic (IL-33 variant lacking exon 3) localization [115]. IL-33 lacking exon 3 possessed constitutive cytokine activity [116]. This study demonstrated the existence of multiple splice variants of IL-33 in different cell types, and that certain transformed cells lacked particular splice variants, suggesting that the transformation of cells may affect the pattern and type of IL-33 that is expressed [115].

### 3.17. Squamous Cell Carcinoma:

IL-33 was shown to play a crucial role in tumor immunosuppression in murine squamous cell carcinoma (SCC) that is mediated by nuclear focal adhesion kinase (FAK). FAK has been implicated in mechanisms of immune escape by inducing the expression of cytokines that inhibit antitumor immunity in the TME. Serrells et al [117] showed that FAK activates a transcriptional network that induces increased nuclear concentration of IL-33 in murine SCC cells that is self-amplifying. Nuclear FAK enhanced the expression of genes encoding IL-33, the CCL5 chemokine, and the soluble ST2 (sST2) receptor. IL-33 then associates with FAK in the nucleus where the IL-33/FAK complex interacts with a network of chromatin modifiers and transcriptional regulators including TAF9, WDR82, and BRD4 that promote the activity of NF-kB which induced the expression of chemokines. There was no secretion of IL-33 detected in the FAK-positive SCC cells. The authors propose that overexpression of sST2 sequesters IL-33 secreted by other cells in the TME to block its stimulatory effect on infiltrating immune cells. Depletion of FAK, IL-33, or sST2 from SCC cells prior to implantation into syngeneic host mice induced tumor regression, unless CD8^+^ T cells were also depleted [117]. The overall effect is tumor immunosuppression through increased stimulation of immunosuppressive regulatory T cells by CCL5 and sequestration of IL-33 by sST2 to block its stimulation of cytotoxic T-cells. Thus, targeting FAK/IL-33-mediated signals may enhance the ability of the patient’s immune system to find and eliminate tumor cells. [117]

A novel mechanism by which IL-33 promotes growth of oral squamous cell carcinoma (OSCC) is by modifying the TME in collaboration with a long non-coding RNA. RNA sequencing analysis identified a lncRNA signature during the transformation of cancer associated fibroblasts (CAFs) from normal fibroblasts (NFs) in the tumor stroma [118]. Notably, a previously uncharacterized lncRNA, FLJ22447 (Lnc-CAF) was highly up-regulated in CAFs. In this model, IL-33 was mainly expressed in the tumor stroma and positively correlated with Lnc-CAF to increase the expression of the CAF markers α-SMA, vimentin, and cadherin. Knockdown of IL-33 impaired the ability of Lnc-CAF to activate stromal fibroblasts and resulted in tumor regression. Mechanistically, Lnc-CAF up-regulated IL-33 levels and prevented its degradation by autophagy-lysosome. In turn, tumor cells further increased Lnc-CAF levels in stromal fibroblasts via exosomal transfer of Lnc-CAF. In patients with OSCC, high Lnc-CAF/IL-33 expression correlated with high TNM stage while high Lnc-CAF expression predicted poor prognosis. *In vivo*, Lnc-CAF knockdown restricted tumor growth and was associated with decreased Ki-67 expression and α-SMA+ CAF in the stroma. Thus, Lnc-CAF/IL-33 promotes OSCC development by modifying the TME through reprogramming of NFs to CAFs in the tumor stroma [118].

### 3.18. Blood Cancers

Myeloproliferative neoplasms (MPNs) include a heterogeneous group of malignant clonal hematopoietic diseases that include BCR-ABL1-negative polycythemia vera (PV), essential thrombocythemia (ET), and primary myelofibrosis (PMF), as well as BCR-ABL1-positive chronic myelogenous leukemia (CML), among others [119]. Although mutations that lead to mis-regulation of critical signaling pathways underlie disease progression in MPNs, several cytokines have been found to be aberrantly expressed [119]. Among these, IL-33 was shown to play a critical role in promoting dysregulated myelopoiesis in a mouse model of MPN. Mice that are homozygous for a mutant allele of inositol polyphosphate-5-phosphatase D (*Inpp5d*) called *styx* (referred to as SHIP mice) recapitulate the pathogenesis of the MPN-like disease in *Inpp5d* knockout mice. Loss of *MyD88* and *Irak4* abrogated disease progression in these mice [119]. Both proteins transduce signals through toll-like receptors (TLRs) implicating microbial infection as a causative agent, and through IL-1, IL-18, and IL-33 signaling pathways. However, only the genetic deletion of *Il-33* was sufficient and necessary to reverse the disease phenotypes and restore normal hematopoiesis [119]. IL-33 from stromal cells stimulated the secretion of cytokines and growth factors by both myeloid and non-hematopoietic cells in bone marrow to restore myeloproliferation in SHIP mice. Increased numbers of IL-33-expressing cells were detected in biopsies from MPN patients, and exogenous IL-33 promoted cytokine and colony production by CD34^+^ MPN stem/progenitor cells from patients [119]. Thus, IL-33 is a critical cytokine in the development and progression of MPN malignancies.

Consistent with these studies, CD34^+^ progenitors from patients with chronic myeloid leukemia were selectively targeted by IL-33. In response to IL-33, these cells exhibited increased expression of ST2, proliferated, and produced additional cytokines, while progenitor cells from healthy individuals did not and were unresponsive to IL-33 [120]. Deregulation of the IL-33/ST2 pathway was a consequence of the tyrosine kinase activity by the BCR-ABL1 oncogene [120]. In xenograft experiments, engraftment of CD34^+^ bone marrow progenitor cells from patients and mice expressing BCR-ABL1 were less efficient in *Il-33* knockout mice as compared to wild type mice, suggesting that IL-33 is necessary for CD34^+^ progenitor growth and maintenance in vivo [120].

Further studies showed that in basophil-like chronic myelogenous leukemia KU812 cells, the ST2 receptor is constitutively expressed and facilitates the production of cytokines, chemokines, and growth factors in response to IL-33 [121]. Binding of IL-33 to ST2 resulted in activation of the NF-κB, JNK, and p38 MAPK but not the ERK1/2 pathways [121]. These cells provided an in vitro, cell line-based model to study the mechanisms of IL-33/ST2 signaling [121].

In other studies, patients with PV and ET, had decreased plasma IL-33 levels that were thought to alter their immune system [122]. Similarly, patients with multiple myeloma also had diminished plasma levels of IL-33 that were thought to contribute to changes in their immune system that lead to increased tumor growth and loss of immune system control [123]. In addition, patients with chronic lymphocytic leukemia (CLL) had lower serum IL-33 levels as compared to normal healthy individuals [124]. Although serum IL-33 levels were notably increased following therapy in CLL patients, the data were not statically significant [124]. In all, the IL-33/ST2 is a critical signaling pathway for the development of MPNs.

### 3.19. Lymphoma

A mouse model of lymphoma was used to test the ability of IL-33 to enhance γδ anti-cancer cell therapy [125]. A prominent subset of γδ cells in peripheral blood express the Vγ9 T cell receptor. These cells can be activated with phosphoantigen (PAgs) to induce rapid proliferation and release pro-inflammatory cytokines and chemokines that mediate cell cytotoxicity against a number of malignancies [126]. Cancer cells that overproduce PAgs such as Burkitt’s lymphomas are especially vulnerable to recognition and killing by Vγ9 T cells [125] making these cells attractive for use in cancer immunotherapy. In clinical trials, the combination of PAgs with IL-2 potently activates Vγ9 T cells resulting in tumor regression in some patients [127,128]. However, this approach has been severely limited by toxicity from IL-12 resulting in fatal capillary leak syndrome [129]. Doualt et al. showed that IL-33 can be used in place of IL-12 to potently induce the proliferation of PAg-activated Vγ9 T cells both in vitro and in vivo which prevented tumor growth in a mouse model of lymphoma [125,130]. Thus, IL-33 can be used to potentiate the efficacy of cell-based anti-cancer immunotherapy without the IL-12 induced cytotoxicity.

### 3.20. Bone Cancer Pain Management

Pain is the most frequent symptom in bone cancer patients that drastically affects quality of life. Using a mouse model of bone cancer wherein 4T1 mammary cancer cells were inoculated inside the medullar region of the hind femur, Zhao et al. [131] reported a role for spinal IL-33/ST2 in bone cancer pain. Mice with bone cancer showed significant indication of pain as measured by assessment of limb use, mechanical allodynia and hot plate test as compared to mice treated with PBS or inoculated with heat killed cancer cells. While all the mice showed normal weight gain over time, tumor bearing mice showed significantly low levels of paw withdrawal mechanical threshold (PWT) by von Frey hair stimulation and paw withdrawal thermal latency (PWL) by hot plate test as compared to the control groups over time, culminating with bone destruction [131]. They showed significantly higher expression of IL-33 and other related cytokines (IL-1β, IL-6, TNF-α) in spinal cords. Moreover, IL-33 was mainly expressed by astrocytes expressing glial fibrillary acidic protein (GFAP) inside the spinal cord. Intrathecal administration of ST2 antibody suppressed the pain behavior, suggesting that IL-33/ST2 might play a critical role in cancer-induced pain and could serve as a putative target in bone cancer pain management [131].

## 4. Concluding Remarks

**Figure 4:**
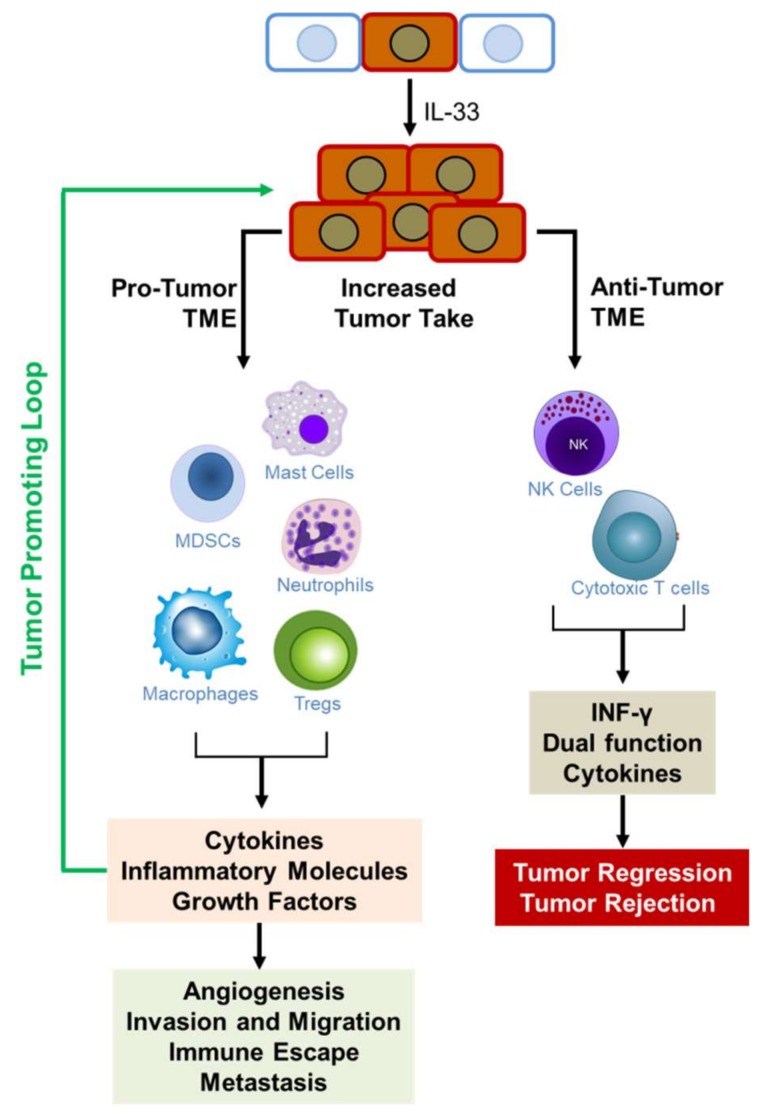
The opposing roles of IL-33/ST2 in cancer. IL-33 promotes increased tumor take of transplanted tumors. Pro-Tumor TME: IL-33 directs the recruitment of pro-inflammatory cells into the tumor microenvironment where they secrete molecules that promote angiogenesis, invasion and metastasis. The secreted molecules can act in paracrine and autocrine mechanisms to create a sustained loop that support continued tumor growth and advancement to malignancy. Anti-Tumor TME: IL-33 directs the recruitment of NK cells and cytotoxic T cells into the TME where they secrete molecules that lead to tumor regression and rejection. Black arrows indicate effect of IL-33 expression on immune cell recruitment, production of cytokines and pro- or anti-inflammatory molecules, and their effect on tumor behavior and phenotype in conditions that promote or block tumor growth. Green arrow indicates a tumor promoting loop mediated by cytokines secreted by immune cells that further activate IL-33 in cancer cells to sustain tumor growth.

IL-33/ST2 signaling is emerging as important pathway in cancer biology. While its roles in inflammatory pathways, immune response, and tissue injury and repair have been extensively studied, its emerging role in cancer pathology is increasingly being elucidated. However, it has been implicated in opposing roles in cancer development (Figure 4). There is abundant evidence indicating that IL-33/ST2 signaling induces the expression of cytokines that orchestrate many aspects of cancer pathology including angiogenesis, invasiveness, immune protection, and metastasis. On the other hand, other data provide evidence that it acts in an anti-tumorigenic role to promote tumor regression and eradication. In both cases, IL-33/ST2 signaling exerts its effect by potently remodeling the tumor microenvironment through recruitment of immune cells that secrete a diverse collection of molecules that enforce their impact on tumor phenotype. Recruitment of mast cells, macrophages, MDSCs, neutrophils, and T_regs_, among others, creates a fertile TME that supports tumor growth and malignancy, and through the secretion of pro-inflammatory cytokines, creates a self-sustaining tumor-promoting loop (see Figure 3). On the other hand, recruitment of NK cells and cytotoxic T cells create an anti-tumorigenic TME that suppresses tumor growth. Figure 4 illustrates the effects of IL-33/ST2 signaling in tumor development. In Table 1, we summarize the pro- and/or anti-tumorigenic effects of IL-33/ST2 in various cancers and indicate the experimental models used to support the observed function as well as the references. IL33/ST2 signaling also appears to be tissue and stage dependent. In all, it is important to understand the mechanisms by which IL-33 impacts the development and malignancy of different types of cancers. This knowledge will facilitate the development of therapies targeting IL-33/ST2 to block cancer progression or as adjuvant therapy to enhance immunotherapies against cancer. This knowledge is also critical in assessing its importance as a biomarker for early diagnosis or determining patient prognosis.

## Figures and Tables

**Table 1:** Summary of data on the effect of IL-33/ST2 signaling on development of different cancers.

Type of Cancer	Pro- or Anti-Tumorigenic Effect on Tumor	Model Used	References
Breast	Pro-tumorigenic	Mouse models	[51,52,54,55,56,57]
		Patient tissues	[56,57,58,59,60,61]
Colon	Pro-tumorigenic	Mouse model	[66,67,68,72,73,74]
		Patient tissue and cell lines	[64,65,72,74]
	Anti-tumorigenic	Mouse model and cell lines	[47,75]
Gastric	Pro-tumorigenic	Mouse model	[78,79]
Hepatocellular	Pro-tumorigenic	Patient sera or plasma, resected patient tissues	[80,81,82,83]
Hepatobiliary	Pro-tumorigenic	Mouse model of CCA Patient tissues	[84]
Pancreatic	Pro-tumorigenic	Cell lines, tissues	[87,88]
Lung	Pro-tumorigenic	Patient samples and cell lines	[90,91,92,93,94]
	Anti-tumorigenic	Patient studies	[95,96,97]
		Mouse models	[76,98,99]
Prostate & kidney	Pro-tumorigenic	Mouse Model	[100]
	Anti-tumorigenic	Patient tissue	[99]
Ovarian	Pro-tumorigenic	Patient tissues and human ovarian cell lines	[101]
Uterine	Pro-tumorigenic	Patient sera	[102,103]
Endometrial	Pro-tumorigenic	Patient sera and tissues	[104]
Cervical	Pro-tumorigenic	Patient tissue and in vitro studies	[105]
Head and Neck	Pro-tumorigenic	Organotypic culture and patient tissues	[106]
Brain	Pro-tumorigenic	Patient tissue	[107]
		Rat model of glioma	[108]
Mouth	Pro-tumorigenic	Human tissues	[109,111]
		Mouse model of salivary gland tumor	[110]
	Anti-tumorigenic	Patient tissue immunohistochemistry	[112]
Skin	Pro-tumorigenic	Mouse and human cell lines	[113]
	Anti-tumorigenic	Mouse models and cell lines	[114]
Squamous cell carcinoma	Pro-tumorigenic	Mouse cell lines and model Cell lines and patient tissues	[117,118]
Blood cancers	Pro-tumorigenic	Mouse model and patient biopsies	[119,120]
		Cell lines	[121]
		Patient plasma and tissues	[122,123,124]
Lymphoma	Pro-tumorigenic	Mouse model and patient tissue	[125,130]
Bone cancer pain	Pro-tumorigenic	Mouse model	[131]

## Abbreviations

IL-33	Interleukin 33
ST2	Suppressor of tumorigenesis
NF-κB	Nuclear factor κB
NK	Natural killer cells
MDSC	Myeloid derived suppressor cells
TME	Tumor microenvironment
HDAC	Histone deacetylase
IL1-RacP	IL1-like receptor accessory protein
MVD	Microvessel density
VEGF	Vascular endothelial growth factor
CRC	Colorectal cancer
NSCLC	Non-small cell lung carcinoma
ELISA	Enzyme linked immunosorbent assay
MPN	Myeloproliferative neoplasms
CAF	Cancer associated fibroblasts

## References

[1] National Center for Health Statistics, Health and Human Services (2016). United States 2016: With Chartbook on Long-Term Trands in Health.

[2] Siegel R.L., Miller K.D., Jemal A. (2018). Cancer Statistics, 2018. CA Cancer J. Clin..

[3] Hanahan D., Weinberg R.A. (2011). Hallmarks of cancer: The next generation. Cell.

[4] Quail D.F., Joyce J.A. (2013). Microenvironmental regulation of tumor progression and metastasis. Nat. Med..

[5] Hanahan D., Coussens L.M. (2012). Accessories to the crime: Functions of cells recruited to the tumor microenvironment. Cancer Cell.

[6] Schmitz J., Owyang A., Oldham E., Song Y., Murphy E., McClanahan T.K., Zurawski G., Moshrefi M., Qin J., Li X. (2005). IL-33, an interleukin-1-like cytokine that signals via the IL-1 receptor-related protein ST2 and induces T helper type 2-associated cytokines. Immunity.

[7] Liew F.Y., Girard J.P., Turnquist H.R. (2016). Interleukin-33 in health and disease. Nat. Rev. Immunol..

[8] Liew F.Y., Pitman N.I., McInnes I.B. (2010). Disease-associated functions of IL-33: The new kid in the IL-1 family. Nat. Rev. Immunol..

[9] Verri W.A., Souto F.O., Vieira S.M., Almeida S.C., Fukada S.Y., Xu D., Alves-Filho J.C., Cunha T.M., Guerrero A.T., Mattos-Guimaraes R.B. (2010). IL-33 induces neutrophil migration in rheumatoid arthritis and is a target of anti-TNF therapy. Ann. Rheum Dis..

[10] Shimizu M., Matsuda A., Yanagisawa K., Hirota T., Akahoshi M., Inomata N., Ebe K., Tanaka K., Sugiura H., Nakashima K. (2005). Functional SNPs in the distal promoter of the ST2 gene are associated with atopic dermatitis. Hum. Mol. Genet..

[11] Hayakawa H., Hayakawa M., Kume A., Tominaga S. (2007). Soluble ST2 blocks interleukin-33 signaling in allergic airway inflammation. J. Biol. Chem..

[12] Nunes T., Bernardazzi C., de Souza H.S. (2014). Interleukin-33 and inflammatory bowel diseases: Lessons from human studies. Mediat. Inflamm..

[13] Sanada S., Hakuno D., Higgins L.J., Schreiter E.R., McKenzie A.N., Lee R.T. (2007). IL-33 and ST2 comprise a critical biomechanically induced and cardioprotective signaling system. J. Clin. Investig..

[14] Miller A.M., Liew F.Y. (2011). The IL-33/ST2 pathway—A new therapeutic target in cardiovascular disease. Pharmacol. Ther..

[15] Kakkar R., Lee R.T. (2008). The IL-33/ST2 pathway: Therapeutic target and novel biomarker. Nat. Rev. Drug Discov..

[16] Moussion C., Ortega N., Girard J.P. (2008). The IL-1-like cytokine IL-33 is constitutively expressed in the nucleus of endothelial cells and epithelial cells in vivo: A novel ‘alarmin’?. PLoS ONE.

[17] Haraldsen G., Balogh J., Pollheimer J., Sponheim J., Kuchler A.M. (2009). Interleukin-33–cytokine of dual function or novel alarmin?. Trends Immunol..

[18] Carriere V., Roussel L., Ortega N., Lacorre D.A., Americh L., Aguilar L., Bouche G., Girard J.P. (2007). IL-33, the IL-1-like cytokine ligand for ST2 receptor, is a chromatin-associated nuclear factor in vivo. Proc. Natl. Acad. Sci. USA.

[19] Roussel L., Erard M., Cayrol C., Girard J.P. (2008). Molecular mimicry between IL-33 and KSHV for attachment to chromatin through the H2A-H2B acidic pocket. EMBO Rep..

[20] Zhang F., Tossberg J.T., Spurlock C.F., Yao S.Y., Aune T.M., Sriram S. (2014). Expression of IL-33 and its epigenetic regulation in Multiple Sclerosis. Ann. Clin. Transl. Neurol..

[21] Ali S., Mohs A., Thomas M., Klare J., Ross R., Schmitz M.L., Martin M.U. (2011). The dual function cytokine IL-33 interacts with the transcription factor NF-kappaB to dampen NF-kappaB-stimulated gene transcription. J. Immunol..

[22] Zhao W., Hu Z. (2010). The enigmatic processing and secretion of interleukin-33. Cell. Mol. Immunol..

[23] Cayrol C., Girard J.P. (2009). The IL-1-like cytokine IL-33 is inactivated after maturation by caspase-1. Proc. Natl. Acad. Sci. USA.

[24] Cayrol C., Girard J.P. (2014). IL-33: An alarmin cytokine with crucial roles in innate immunity, inflammation and allergy. Curr. Opin. Immunol..

[25] Bessa J., Meyer C.A., de Vera Mudry M.C., Schlicht S., Smith S.H., Iglesias A., Cote-Sierra J. (2014). Altered subcellular localization of IL-33 leads to non-resolving lethal inflammation. J. Autoimmun..

[26] Talabot-Ayer D., Lamacchia C., Gabay C., Palmer G. (2009). Interleukin-33 is biologically active independently of caspase-1 cleavage. J. Biol. Chem..

[27] Ali S., Nguyen D.Q., Falk W., Martin M.U. (2010). Caspase 3 inactivates biologically active full length interleukin-33 as a classical cytokine but does not prohibit nuclear translocation. Biochem. Biophys. Res. Commun..

[28] Luthi A.U., Cullen S.P., McNeela E.A., Duriez P.J., Afonina I.S., Sheridan C., Brumatti G., Taylor R.C., Kersse K., Vandenabeele P. (2009). Suppression of interleukin-33 bioactivity through proteolysis by apoptotic caspases. Immunity.

[29] Lefrancais E., Roga S., Gautier V., Gonzalez-de-Peredo A., Monsarrat B., Girard J.P., Cayrol C. (2012). IL-33 is processed into mature bioactive forms by neutrophil elastase and cathepsin G. Proc. Natl. Acad. Sci. USA.

[30] Lefrancais E., Duval A., Mirey E., Roga S., Espinosa E., Cayrol C., Girard J.P. (2014). Central domain of IL-33 is cleaved by mast cell proteases for potent activation of group-2 innate lymphoid cells. Proc. Natl. Acad. Sci. USA.

[31] Lefrancais E., Cayrol C. (2012). Mechanisms of IL-33 processing and secretion: Differences and similarities between IL-1 family members. Eur. Cytokine Netw..

[32] Tominaga S. (1989). A putative protein of a growth specific cDNA from BALB/c-3T3 cells is highly similar to the extracellular portion of mouse interleukin 1 receptor. FEBS Lett..

[33] Tominaga S., Yokota T., Yanagisawa K., Tsukamoto T., Takagi T., Tetsuka T. (1992). Nucleotide sequence of a complementary DNA for human ST2. Biochim. Biophys. Acta.

[34] Yanagisawa K., Takagi T., Tsukamoto T., Tetsuka T., Tominaga S. (1993). Presence of a novel primary response gene ST2L, encoding a product highly similar to the interleukin 1 receptor type 1. FEBS Lett..

[35] Hardman C., Ogg G. (2016). Interleukin-33, friend and foe in type-2 immune responses. Curr. Opin. Immunol..

[36] Bergers G., Reikerstorfer A., Braselmann S., Graninger P., Busslinger M. (1994). Alternative promoter usage of the Fos-responsive gene Fit-1 generates mRNA isoforms coding for either secreted or membrane-bound proteins related to the IL-1 receptor. EMBO J..

[37] Tominaga S., Kuroiwa K., Tago K., Iwahana H., Yanagisawa K., Komatsu N. (1999). Presence and expression of a novel variant form of ST2 gene product in human leukemic cell line UT-7/GM. Biochem. Biophys. Res. Commun..

[38] Tago K., Noda T., Hayakawa M., Iwahana H., Yanagisawa K., Yashiro T., Tominaga S. (2001). Tissue distribution and subcellular localization of a variant form of the human ST2 gene product, ST2V. Biochem. Biophys. Res. Commun..

[39] Iwahana H., Hayakawa M., Kuroiwa K., Tago K., Yanagisawa K., Noji S., Tominaga S. (2004). Molecular cloning of the chicken ST2 gene and a novel variant form of the ST2 gene product, ST2LV. Biochim. Biophys. Acta.

[40] Oshikawa K., Yanagisawa K., Tominaga S., Sugiyama Y. (2002). Expression and function of the ST2 gene in a murine model of allergic airway inflammation. Clin. Exp. Allergy.

[41] Iwahana H., Yanagisawa K., Ito-Kosaka A., Kuroiwa K., Tago K., Komatsu N., Katashima R., Itakura M., Tominaga S. (1999). Different promoter usage and multiple transcription initiation sites of the interleukin-1 receptor-related human ST2 gene in UT-7 and TM12 cells. Eur. J. Biochem..

[42] Baba Y., Maeda K., Yashiro T., Inage E., Kasakura K., Suzuki R., Niyonsaba F., Hara M., Tanabe A., Ogawa H. (2012). GATA2 is a critical transactivator for the human IL1RL1/ST2 promoter in mast cells/basophils: Opposing roles for GATA2 and GATA1 in human IL1RL1/ST2 gene expression. J. Biol. Chem..

[43] Akimoto M., Takenaga K. (2018). Role of the IL-33/ST2L axis in colorectal cancer progression. Cell. Immunol..

[44] Millar N.L., O’Donnell C., McInnes I.B., Brint E. (2017). Wounds that heal and wounds that don’t—The role of the IL-33/ST2 pathway in tissue repair and tumorigenesis. Semin. Cell Dev. Biol..

[45] Funakoshi-Tago M., Tago K., Hayakawa M., Tominaga S., Ohshio T., Sonoda Y., Kasahara T. (2008). TRAF6 is a critical signal transducer in IL-33 signaling pathway. Cell. Signal..

[46] Milovanovic M., Volarevic V., Radosavljevic G., Jovanovic I., Pejnovic N., Arsenijevic N., Lukic M.L. (2012). IL-33/ST2 axis in inflammation and immunopathology. Immunol. Res..

[47] O’Donnell C., Mahmoud A., Keane J., Murphy C., White D., Carey S., O’Riordain M., Bennett M.W., Brint E., Houston A. (2016). An antitumorigenic role for the IL-33 receptor, ST2L, in colon cancer. Br. J. Cancer.

[48] Zhao J., Wei J., Mialki R.K., Mallampalli D.F., Chen B.B., Coon T., Zou C., Mallampalli R.K., Zhao Y. (2012). F-box protein FBXL19-mediated ubiquitination and degradation of the receptor for IL-33 limits pulmonary inflammation. Nat. Immunol..

[49] Kumar S., Tzimas M.N., Griswold D.E., Young P.R. (1997). Expression of ST2, an interleukin-1 receptor homologue, is induced by proinflammatory stimuli. Biochem. Biophys. Res. Commun..

[50] Cohen E.S., Scott I.C., Majithiya J.B., Rapley L., Kemp B.P., England E., Rees D.G., Overed-Sayer C.L., Woods J., Bond N.J. (2015). Oxidation of the alarmin IL-33 regulates ST2-dependent inflammation. Nat. Commun..

[51] Jovanovic I., Radosavljevic G., Mitrovic M., Juranic V.L., McKenzie A.N., Arsenijevic N., Jonjic S., Lukic M.L. (2011). ST2 deletion enhances innate and acquired immunity to murine mammary carcinoma. Eur. J. Immunol..

[52] Jovanovic I.P., Pejnovic N.N., Radosavljevic G.D., Arsenijevic N.N., Lukic M.L. (2012). IL-33/ST2 axis in innate and acquired immunity to tumors. Oncoimmunology.

[53] Gillibert-Duplantier J., Duthey B., Sisirak V., Salaun D., Gargi T., Tredan O., Finetti P., Bertucci F., Birnbaum D., Bendriss-Vermare N. (2012). Gene expression profiling identifies sST2 as an effector of ErbB2-driven breast carcinoma cell motility, associated with metastasis. Oncogene.

[54] Jovanovic I.P., Pejnovic N.N., Radosavljevic G.D., Pantic J.M., Milovanovic M.Z., Arsenijevic N.N., Lukic M.L. (2014). Interleukin-33/ST2 axis promotes breast cancer growth and metastases by facilitating intratumoral accumulation of immunosuppressive and innate lymphoid cells. Int. J. Cancer.

[55] Xiao P., Wan X., Cui B., Liu Y., Qiu C., Rong J., Zheng M., Song Y., Chen L., He J. (2016). Interleukin 33 in tumor microenvironment is crucial for the accumulation and function of myeloid-derived suppressor cells. Oncoimmunology.

[56] Milosavljevic M.Z., Jovanovic I.P., Pejnovic N.N., Mitrovic S.L., Arsenijevic N.N., Simovic Markovic B.J., Lukic M.L. (2016). Deletion of IL-33R attenuates VEGF expression and enhances necrosis in mammary carcinoma. Oncotarget.

[57] Kim J.Y., Lim S.C., Kim G., Yun H.J., Ahn S.G., Choi H.S. (2015). Interleukin-33/ST2 axis promotes epithelial cell transformation and breast tumorigenesis via upregulation of COT activity. Oncogene.

[58] Lu D.P., Zhou X.Y., Yao L.T., Liu C.G., Ma W., Jin F., Wu Y.F. (2014). Serum soluble ST2 is associated with ER-positive breast cancer. BMC Cancer.

[59] Yang Z.P., Ling D.Y., Xie Y.H., Wu W.X., Li J.R., Jiang J., Zheng J.L., Fan Y.H., Zhang Y. (2015). The Association of Serum IL-33 and sST2 with Breast Cancer. Dis. Mark..

[60] Liu J., Shen J.X., Hu J.L., Huang W.H., Zhang G.J. (2014). Significance of interleukin-33 and its related cytokines in patients with breast cancers. Front. Immunol..

[61] Jafarzadeh A., Minaee K., Farsinejad A.R., Nemati M., Khosravimashizi A., Daneshvar H., Mohammadi M.M., Sheikhi A., Ghaderi A. (2015). Evaluation of the circulating levels of IL-12 and IL-33 in patients with breast cancer: Influences of the tumor stages and cytokine gene polymorphisms. Iran. J. Basic Med. Sci..

[62] Yigitbasi M.R., Guntas G., Atak T., Sonmez C., Yalman H., Uzun H. (2017). The Role of Interleukin-33 as an Inflammatory Marker in Differential Diagnosis of Idiopathic Granulomatous Mastitis and Breast Cancer. J. Investig. Surg.

[63] American Cancer Society, American Cancer Society (2018). Cancer Facts and Figures 2018.

[64] Cui G., Qi H., Gundersen M.D., Yang H., Christiansen I., Sorbye S.W., Goll R., Florholmen J. (2015). Dynamics of the IL-33/ST2 network in the progression of human colorectal adenoma to sporadic colorectal cancer. Cancer Immunol. Immunother..

[65] Liu X., Zhu L., Lu X., Bian H., Wu X., Yang W., Qin Q. (2014). IL-33/ST2 pathway contributes to metastasis of human colorectal cancer. Biochem. Biophys. Res. Commun..

[66] Maywald R.L., Doerner S.K., Pastorelli L., De Salvo C., Benton S.M., Dawson E.P., Lanza D.G., Berger N.A., Markowitz S.D., Lenz H.J. (2015). IL-33 activates tumor stroma to promote intestinal polyposis. Proc. Natl. Acad. Sci. USA.

[67] Mertz K.D., Mager L.F., Wasmer M.H., Thiesler T., Koelzer V.H., Ruzzante G., Joller S., Murdoch J.R., Brummendorf T., Genitsch V. (2016). The IL-33/ST2 pathway contributes to intestinal tumorigenesis in humans and mice. Oncoimmunology.

[68] Zhang Y., Davis C., Shah S., Hughes D., Ryan J.C., Altomare D., Pena M.M. (2017). IL-33 promotes growth and liver metastasis of colorectal cancer in mice by remodeling the tumor microenvironment and inducing angiogenesis. Mol. Carcinog..

[69] Gounaris E., Erdman S.E., Restaino C., Gurish M.F., Friend D.S., Gounari F., Lee D.M., Zhang G., Glickman J.N., Shin K. (2007). Mast cells are an essential hematopoietic component for polyp development. Proc. Natl. Acad. Sci. USA.

[70] Khazaie K., Blatner N.R., Khan M.W., Gounari F., Gounaris E., Dennis K., Bonertz A., Tsai F.N., Strouch M.J., Cheon E. (2011). The significant role of mast cells in cancer. Cancer Metast. Rev..

[71] Saadalla A.M., Osman A., Gurish M.F., Dennis K.L., Blatner N.R., Pezeshki A., McNagny K.M., Cheroutre H., Gounari F., Khazaie K. (2018). Mast cells promote small bowel cancer in a tumor stage-specific and cytokine-dependent manner. Proc. Natl. Acad. Sci. USA.

[72] Akimoto M., Maruyama R., Takamaru H., Ochiya T., Takenaga K. (2016). Soluble IL-33 receptor sST2 inhibits colorectal cancer malignant growth by modifying the tumour microenvironment. Nat. Commun..

[73] Fang M., Li Y., Huang K., Qi S., Zhang J., Zgodzinski W., Majewski M., Wallner G., Gozdz S., Macek P. (2017). IL33 Promotes Colon Cancer Cell Stemness via JNK Activation and Macrophage Recruitment. Cancer Res..

[74] He Z., Chen L., Souto F.O., Canasto-Chibuque C., Bongers G., Deshpande M., Harpaz N., Ko H.M., Kelley K., Furtado G.C. (2017). Epithelial-derived IL-33 promotes intestinal tumorigenesis in Apc (Min/+) mice. Sci. Rep..

[75] Eissmann M.F., Dijkstra C., Wouters M.A., Baloyan D., Mouradov D., Nguyen P.M., Davalos-Salas M., Putoczki T.L., Sieber O.M., Mariadason J.M. (2018). Interleukin 33 Signaling Restrains Sporadic Colon Cancer in an Interferon-gamma-Dependent Manner. Cancer Immunol. Res..

[76] Gao K., Li X., Zhang L., Bai L., Dong W., Gao K., Shi G., Xia X., Wu L., Zhang L. (2013). Transgenic expression of IL-33 activates CD8(+) T cells and NK cells and inhibits tumor growth and metastasis in mice. Cancer Lett..

[77] Gao X., Wang X., Yang Q., Zhao X., Wen W., Li G., Lu J., Qin W., Qi Y., Xie F. (2015). Tumoral expression of IL-33 inhibits tumor growth and modifies the tumor microenvironment through CD8^+^ T and NK cells. J. Immunol..

[78] Meyer A.R., Goldenring J.R. (2018). Injury, repair, inflammation and metaplasia in the stomach. J. Physiol..

[79] Petersen C.P., Meyer A.R., De Salvo C., Choi E., Schlegel C., Petersen A., Engevik A.C., Prasad N., Levy S.E., Peebles R.S. (2018). A signalling cascade of IL-33 to IL-13 regulates metaplasia in the mouse stomach. Gut.

[80] Bergis D., Kassis V., Ranglack A., Koeberle V., Piiper A., Kronenberger B., Zeuzem S., Waidmann O., Radeke H.H. (2013). High Serum Levels of the Interleukin-33 Receptor Soluble ST2 as a Negative Prognostic Factor in Hepatocellular Carcinoma. Transl. Oncol..

[81] Brunner S.M., Rubner C., Kesselring R., Martin M., Griesshammer E., Ruemmele P., Stempfl T., Teufel A., Schlitt H.J., Fichtner-Feigl S. (2015). Tumor-infiltrating, interleukin-33-producing effector-memory CD8^+^ T cells in resected hepatocellular carcinoma prolong patient survival. Hepatology.

[82] Zhang P., Liu X.K., Chu Z., Ye J.C., Li K.L., Zhuang W.L., Yang D.J., Jiang Y.F. (2012). Detection of interleukin-33 in serum and carcinoma tissue from patients with hepatocellular carcinoma and its clinical implications. J. Int. Med. Res..

[83] Wei Z.H., Li Y.Y., Huang S.Q., Tan Z.Q. (2018). Genetic variants in IL-33/ST2 pathway with the susceptibility to hepatocellular carcinoma in a Chinese population. Cytokine.

[84] Yamada D., Rizvi S., Razumilava N., Bronk S.F., Davila J.I., Champion M.D., Borad M.J., Bezerra J.A., Chen X., Gores G.J. (2015). IL-33 facilitates oncogene-induced cholangiocarcinoma in mice by an interleukin-6-sensitive mechanism. Hepatology.

[85] Li J., Razumilava N., Gores G.J., Walters S., Mizuochi T., Mourya R., Bessho K., Wang Y.H., Glaser S.S., Shivakumar P. (2014). Biliary repair and carcinogenesis are mediated by IL-33-dependent cholangiocyte proliferation. J. Clin. Investig..

[86] Centers for Disease Control and Prevention and National Cancer Institute, U.S. Department of Health and Human Services (2017). United States Cancer Statistics: 1999–2014 Incidence and Mortality Web-Based Report.

[87] Masamune A., Watanabe T., Kikuta K., Satoh K., Kanno A., Shimosegawa T. (2010). Nuclear expression of interleukin-33 in pancreatic stellate cells. Am. J. Physiol. Gastrointest. Liver Physiol..

[88] Schmieder A., Multhoff G., Radons J. (2012). Interleukin-33 acts as a pro-inflammatory cytokine and modulates its receptor gene expression in highly metastatic human pancreatic carcinoma cells. Cytokine.

[89] Naumnik W., Naumnik B., Niewiarowska K., Ossolinska M., Chyczewska E. (2012). Novel cytokines: IL-27, IL-29, IL-31 and IL-33. Can they be useful in clinical practice at the time diagnosis of lung cancer?. Exp. Oncol..

[90] Hu L.A., Fu Y., Zhang D.N., Zhang J. (2013). Serum IL-33 as a diagnostic and prognostic marker in non-small cell lung cancer. Asian Pac. J. Cancer Prev..

[91] Wang C., Chen Z., Bu X., Han Y., Shan S., Ren T., Song W. (2016). IL-33 signaling fuels outgrowth and metastasis of human lung cancer. Biochem. Biophys. Res. Commun..

[92] Yang Z., Gao X., Wang J., Xu L., Zheng Y., Xu Y. (2018). Interleukin-33 enhanced the migration and invasiveness of human lung cancer cells. Onco Targets Ther..

[93] Wang K., Shan S., Yang Z., Gu X., Wang Y., Wang C., Ren T. (2017). IL-33 blockade suppresses tumor growth of human lung cancer through direct and indirect pathways in a preclinical model. Oncotarget.

[94] Sun M., Bai Y., Zhao S., Liu X., Gao Y., Wang L., Liu B., Ma D., Ma C. (2018). Gram-negative bacteria facilitate tumor progression through TLR4/IL-33 pathway in patients with non-small-cell lung cancer. Oncotarget.

[95] Kim M.S., Kim E., Heo J.S., Bae D.J., Lee J.U., Lee T.H., Lee H.J., Chang H.S., Park J.S., Jang A.S. (2015). Circulating IL-33 level is associated with the progression of lung cancer. Lung Cancer.

[96] Akimoto M., Hayashi J.I., Nakae S., Saito H., Takenaga K. (2016). Interleukin-33 enhances programmed oncosis of ST2L-positive low-metastatic cells in the tumour microenvironment of lung cancer. Cell Death Dis..

[97] Yang M., Feng Y., Yue C., Xu B., Chen L., Jiang J., Lu B., Zhu Y. (2018). Lower expression level of IL-33 is associated with poor prognosis of pulmonary adenocarcinoma. PLoS ONE.

[98] Saranchova I., Han J., Zaman R., Arora H., Huang H., Fenninger F., Choi K.B., Munro L., Pfeifer C.G., Welch I. (2018). Type 2 Innate Lymphocytes Actuate Immunity Against Tumours and Limit Cancer Metastasis. Sci. Rep..

[99] Saranchova I., Han J., Huang H., Fenninger F., Choi K.B., Munro L., Pfeifer C., Welch I., Wyatt A.W., Fazli L. (2016). Discovery of a Metastatic Immune Escape Mechanism Initiated by the Loss of Expression of the Tumour Biomarker Interleukin-33. Sci. Rep..

[100] Ravichandran K., Holditch S., Brown C.N., Wang Q., Ozkok A., Weiser-Evans M.C., Nemenoff R., Miyazaki M., Thiessen-Philbrook H., Parikh C.R. (2018). IL-33 deficiency slows cancer growth but does not protect against cisplatin-induced AKI in mice with cancer. Am. J. Physiol. Renal Physiol..

[101] Tong X., Barbour M., Hou K., Gao C., Cao S., Zheng J., Zhao Y., Mu R., Jiang H.R. (2016). Interleukin-33 predicts poor prognosis and promotes ovarian cancer cell growth and metastasis through regulating ERK and JNK signaling pathways. Mol. Oncol..

[102] Santulli P., Even M., Chouzenoux S., Millischer A.E., Borghese B., de Ziegler D., Batteux F., Chapron C. (2013). Profibrotic interleukin-33 is correlated with uterine leiomyoma tumour burden. Hum. Reprod..

[103] Santulli P., Borghese B., Chouzenoux S., Vaiman D., Borderie D., Streuli I., Goffinet F., de Ziegler D., Weill B., Batteux F. (2012). Serum and peritoneal interleukin-33 levels are elevated in deeply infiltrating endometriosis. Hum. Reprod..

[104] Zeng X., Zhang Z., Gao Q.Q., Wang Y.Y., Yu X.Z., Zhou B., Xi M.R. (2016). Clinical Significance of Serum Interleukin-31 and Interleukin-33 Levels in Patients of Endometrial Cancer: A Case Control Study. Dis. Mark..

[105] Wang L., Li H., Liang F., Hong Y., Jiang S., Xiao L. (2014). Examining IL-33 expression in the cervix of HPV-infected patients: A preliminary study comparing IL-33 levels in different stages of disease and analyzing its potential association with IFN-gamma. Med. Oncol..

[106] Chen S.F., Nieh S., Jao S.W., Wu M.Z., Liu C.L., Chang Y.C., Lin Y.S. (2013). The paracrine effect of cancer-associated fibroblast-induced interleukin-33 regulates the invasiveness of head and neck squamous cell carcinoma. J. Pathol..

[107] Gramatzki D., Frei K., Cathomas G., Moch H., Weller M., Mertz K.D. (2016). Interleukin-33 in human gliomas: Expression and prognostic significance. Oncol. Lett..

[108] Fang K.M., Yang C.S., Lin T.C., Chan T.C., Tzeng S.F. (2014). Induced interleukin-33 expression enhances the tumorigenic activity of rat glioma cells. Neuro Oncol..

[109] Tada H., Matsuyama T., Nishioka T., Hagiwara M., Kiyoura Y., Shimauchi H., Matsushita K. (2016). Porphyromonas gingivalis Gingipain-Dependently Enhances IL-33 Production in Human Gingival Epithelial Cells. PLoS ONE.

[110] Mishra R., Polic B., Welsh R.M., Szomolanyi-Tsuda E. (2013). Inflammatory cytokine-mediated evasion of virus-induced tumors from NK cell control. J. Immunol..

[111] Ishikawa K., Yagi-Nakanishi S., Nakanishi Y., Kondo S., Tsuji A., Endo K., Wakisaka N., Murono S., Yoshizaki T. (2014). Expression of interleukin-33 is correlated with poor prognosis of patients with squamous cell carcinoma of the tongue. Auris Nasus Larynx.

[112] Rossle M., Cathomas G., Bonapace L., Sachs M., Dehler S., Storz M., Huber G., Moch H., Junt T., Mertz K.D. (2016). Interleukin-33 Expression Indicates a Favorable Prognosis in Malignant Salivary Gland Tumors. Int. J. Surg. Pathol..

[113] Byrne S.N., Beaugie C., O’Sullivan C., Leighton S., Halliday G.M. (2011). The immune-modulating cytokine and endogenous Alarmin interleukin-33 is upregulated in skin exposed to inflammatory UVB radiation. Am. J. Pathol..

[114] Bergot A.S., Monnet N., Le Tran S., Mittal D., Al-Kouba J., Steptoe R.J., Grimbaldeston M.A., Frazer I.H., Wells J.W. (2015). HPV16 E7 expression in skin induces TSLP secretion, type 2 ILC infiltration and atopic dermatitis-like lesions. Immunol. Cell Biol..

[115] Tsuda H., Komine M., Karakawa M., Etoh T., Tominaga S., Ohtsuki M. (2012). Novel splice variants of IL-33: Differential expression in normal and transformed cells. J. Investig. Dermatol..

[116] Hong J., Bae S., Jhun H., Lee S., Choi J., Kang T., Kwak A., Hong K., Kim E., Jo S. (2011). Identification of Constitutively Active Interleukin 33 (IL-33) Splice Variant. J. Biol. Chem..

[117] Serrels B., McGivern N., Canel M., Byron A., Johnson S.C., McSorley H.J., Quinn N., Taggart D., Von Kreigsheim A., Anderton S.M. (2017). IL-33 and ST2 mediate FAK-dependent antitumor immune evasion through transcriptional networks. Sci Signal.

[118] Ding L., Ren J., Zhang D., Li Y., Huang X., Hu Q., Wang H., Song Y., Ni Y., Hou Y. (2018). A novel stromal lncRNA signature reprograms fibroblasts to promote the growth of oral squamous cell carcinoma via LncRNA-CAF/interleukin-33. Carcinogenesis.

[119] Mager L.F., Riether C., Schurch C.M., Banz Y., Wasmer M.H., Stuber R., Theocharides A.P., Li X., Xia Y., Saito H. (2015). IL-33 signaling contributes to the pathogenesis of myeloproliferative neoplasms. J. Clin. Investig..

[120] Levescot A., Flamant S., Basbous S., Jacomet F., Feraud O., Anne Bourgeois E., Bonnet M.L., Giraud C., Roy L., Barra A. (2014). BCR-ABL-induced deregulation of the IL-33/ST2 pathway in CD34^+^ progenitors from chronic myeloid leukemia patients. Cancer Res..

[121] Tare N., Li H., Morschauser A., Cote-Sierra J., Ju G., Renzetti L., Lin T.A. (2010). KU812 cells provide a novel in vitro model of the human IL-33/ST2L axis: Functional responses and identification of signaling pathways. Exp. Cell Res..

[122] Gangemi S., Allegra A., Profita M., Saitta S., Gerace D., Bonanno A., Alonci A., Petrungaro A., Russo S., Musolino C. (2013). Decreased plasma levels of IL-33 could contribute to the altered function of Th2 lymphocytes in patients with polycythemia vera and essential thrombocythemia. Cancer Investig..

[123] Musolino C., Allegra A., Profita M., Alonci A., Saitta S., Russo S., Bonanno A., Innao V., Gangemi S. (2013). Reduced IL-33 plasma levels in multiple myeloma patients are associated with more advanced stage of disease. Br. J. Haematol..

[124] Musolino C., Allegra A., Profita M., Alonci A., Saitta S., Bonanno A., Gerace D., Calabro L., Gangemi S. (2014). Reduction in IL-33 plasma levels might be involved in T cell dysregulation in chronic lymphocytic leukemia. Acta Haematol..

[125] Duault C., Betous D., Bezombes C., Roga S., Cayrol C., Girard J.P., Fournie J.J., Poupot M. (2017). IL-33-expanded human Vgamma9Vdelta2 T cells have anti-lymphoma effect in a mouse tumor model. Eur. J. Immunol..

[126] Kabelitz D., Wesch D., Pitters E., Zoller M. (2004). Characterization of tumor reactivity of human V gamma 9V delta 2 gamma delta T cells in vitro and in SCID mice in vivo. J. Immunol..

[127] Casetti R., Perretta G., Taglioni A., Mattei M., Colizzi V., Dieli F., D’Offizi G., Malkovsky M., Poccia F. (2005). Drug-induced expansion and differentiation of V gamma 9V delta 2 T cells in vivo: The role of exogenous IL-2. J. Immunol..

[128] Sicard H., Ingoure S., Luciani B., Serraz C., Fournie J.J., Bonneville M., Tiollier J., Romagne F. (2005). In vivo immunomanipulation of V gamma 9V delta 2 T cells with a synthetic phosphoantigen in a preclinical nonhuman primate model. J. Immunol..

[129] Zloza A., Dharmadhikari N.D., Huelsmann E.J., Broucek J.R., Hughes T., Kohlhapp F.J., Kaufman H.L. (2017). Low-dose interleukin-2 impairs host anti-tumor immunity and inhibits therapeutic responses in a mouse model of melanoma. Cancer Immunol. Immunother..

[130] Duault C., Franchini D.M., Familliades J., Cayrol C., Roga S., Girard J.P., Fournie J.J., Poupot M. (2016). TCRVgamma9 gammadelta T Cell Response to IL-33: A CD4 T Cell-Dependent Mechanism. J. Immunol..

[131] Zhao J., Zhang H., Liu S.B., Han P., Hu S., Li Q., Wang Z.F., Mao-Ying Q.L., Chen H.M., Jiang J.W. (2013). Spinal interleukin-33 and its receptor ST2 contribute to bone cancer-induced pain in mice. Neuroscience.

